# Radiotherapy in the preoperative neoadjuvant treatment of locally advanced rectal cancer

**DOI:** 10.3389/fonc.2023.1300535

**Published:** 2023-11-23

**Authors:** Zhen Yu, Yuying Hao, Yuhua Huang, Ling Ling, Xigang Hu, Simiao Qiao

**Affiliations:** Department of Radiation Oncology, Zhujiang Hospital, Southern Medical University, Guangzhou, China

**Keywords:** locally advanced rectal cancer, neoadjuvant treatment, total neoadjuvant treatment, radiotherapy, immunotherapy, targeted therapy

## Abstract

Radiotherapy and chemotherapy are effective treatments for patients with locally advanced rectal cancer (LARC) and can significantly improve the likelihood of R0 resection. Radiotherapy can be used as a local treatment to reduce the size of the tumor, improve the success rate of surgery and reduce the residual cancer cells after surgery. Early chemotherapy can also downgrade the tumor and eliminate micrometastases throughout the body, reducing the risk of recurrence and metastasis. The advent of neoadjuvant concurrent radiotherapy (nCRT) and total neoadjuvant treatment (TNT) has brought substantial clinical benefits to patients with LARC. Even so, given increasing demand for organ preservation and quality of life and the disease becoming increasingly younger in its incidence profile, there is a need to further explore new neoadjuvant treatment options to further improve tumor remission rates and provide other opportunities for patients to choose watch-and-wait (W&W) strategies that avoid surgery. Targeted drugs and immunologic agents (ICIs) have shown good efficacy in patients with advanced rectal cancer but have not been commonly used in neoadjuvant therapy for patients with LARC. In this paper, we review several aspects of neoadjuvant therapy, including radiation therapy and chemotherapy drugs, immune drugs and targeted drugs used in combination with neoadjuvant therapy, with the aim of providing direction and thoughtful perspectives for LARC clinical treatment and research trials.

## 1 Introduction

Rectal cancer is a common malignant tumor of the gastrointestinal tract. The development of rectal cancer is influenced by many factors, especially heredity, dietary habits, obesity, smoking and other factors, and its incidence and mortality rate are increasing yearly. At the same time, the incidence of rectal cancer is trending up in younger people. This issue is attracting increased attention (1). Early rectal cancer can be radically resected by surgery to achieve a good prognosis; however, due to the limitations of the pelvic anatomy, locally advanced rectal cancer (LARC) requires neoadjuvant chemoradiotherapy (nCRT) or total neoadjuvant therapy (TNT) to downgrade the tumor followed by total rectal mesenteric excision (TME) to ensure good R0 resection and anal preservation rates. A small percentage of patients who achieve pathological complete response (pCR) or clinical complete response (cCR) have an opportunity to avoid surgery and adopt a watch-and-wait strategy (W&W) (2–5).

Although LARC patients have better tumor stage reduction with nCRT, the pCR rate has been maintained at a low level. With the continuous advancement of radiotherapy technology, the efficacy of radiotherapy has been improving, and in addition to long-course simultaneous radiotherapy, short-course radiotherapy (SCRT) has again become one of the standard treatment options in recent years (6). At the same time, immunologic and targeted agents have also achieved great success in various solid tumors, which brings new hope for those patients who are not sensitive to chemotherapy; therefore, an increasing number of studies have used different types of drugs in combination with radiotherapy for neoadjuvant treatment to further improve the tumor stage reduction rate and pCR rate. Although the theory is that more intense regimens may increase clinical benefit and toxicity, this has not been found to be the case in many clinical studies. In this paper, we summarize the clinical studies on neoadjuvant treatment of LARC with a combination of radiotherapy and different drugs to provide help and considerations for clinical treatment.

## 2 Radiotherapy

In 1997, a Swedish research group first found that neoadjuvant radiotherapy could substantially reduce the local recurrence rate (LRR) of patients (7). nCRT has now become the standard of care for patients with LARC (8). Neoadjuvant radiotherapy is usually chosen at a dose of 45-50.4 Gy/25-28 F or 25 Gy/5 F (6, 9), but increasing the dose of radiotherapy, expanding the range of irradiation, and changing dose splitting and other modalities to further improve the benefit are unclear.

### 2.1 Neoadjuvant radiotherapy

In the 1990s, 1168 LARC patients were enrolled in a Swedish study and randomly assigned to a preoperative radiotherapy group (n=583) and a surgery-only group (n=585). Short-course radiotherapy (SCRT) increased the incidence of surgical complications and gastrointestinal and urinary toxicity (10). After 5 years of follow-up, however, patients in the SCRT group were found to have better LRR (11% vs. 27%) and 5-year overall survival (58% vs. 48%) than those in the surgery-only group (7), and after up to 13 years of follow-up, patients in the SCRT group had significantly better tumor-specific survival (72% v 62%) and LRR (9% vs. 26%) than those in the surgery-only group (11). A study conducted in the Netherlands similarly found that although the preoperative use of SCRT for TME resulted in more adverse events, its LRR and tumor-specific survival rates were superior to those of patients undergoing surgery alone after 12 years of follow-up (12–14, 2005). This finding was also validated in a study by Sebag-Montefiore, Stephens (15), where improvements were seen in LRR (4.7 vs. 11.5%) and disease-free survival (DFS) (73.6 vs. 66.7%) after preoperative use of neoadjuvant SCRT compared to postoperative chemoradiotherapy.

The German CAO/ARO/AIO-94 study, which first proposed preoperative fluorouracil synchronized chemoradiotherapy, showed improved tumor stage reduction rates and LRR, increased anus preservation rates and lower long-term recurrence rates (16, 2012). Another CAO/ARO/AIO-04 study in Germany showed that the addition of oxaliplatin (OX) to 5-FU-RT significantly improved the DFS of patients at 3 years (17). These studies provide strong evidence for nCRT to become a standard of care.

Although preoperative SCRT improves the clinical benefit of patients compared to surgery alone, it may lead to poor tumor regression due to its shorter treatment period (18). Bujko, Nowacki (19, 2005, 2006) first compared the efficacy of SCRT and nCRT and showed that although long-course radiotherapy did not improve the patient anal preservation rate and had a higher incidence of short-term ≥ grade 3 adverse events, there was a clear advantage in tumor stage reduction and pCR and no difference in long-term toxicity between the two groups after 4 years of follow-up. The TROG trial subsequently built on the Bujko, Nowacki (20) and Ngan, Burmeister (21) trial with more stringent control and allocation of enrolled patients and found that patients with nCRT had better tumor stage reduction and pCR rates than those with SCRT, but there were no significant differences in recurrence rates, 5-year overall survival, or long-term toxicity.

Although nCRT has become the standard treatment modality for LARC, its longer treatment period, poorer patient tolerance and higher treatment costs remain to be addressed, and the regimen does not provide a high pCR rate and survival benefit, so exploring the best treatment option remains a key issue in the clinical management of LARC.

### 2.2 Dose increase

In recent years, there has been an increasing clinical demand for pCR rates and anal preservation rates, but since nCRT can only provide 15-20% pCR rates (22), some studies have explored whether increasing the dose of radiotherapy can improve pCR rates and anal preservation rates.

Jakobsen, Mortensen (23) added 5 Gy of brachytherapy to 60 Gy/30 F in 50 patients with T3 rectal adenocarcinoma and 27% each of patients achieving TRG 1 and 2. They then performed another trial comparing the efficacy in the standard group (50.4 Gy/28 F, n=117) and the dose-enhanced group (50.4 Gy/28 F + 10 Gy/2 F brachytherapy, n=114) and found that the addition of endorectal brachytherapy increased the primary remission rate by 1.5-fold despite not increasing the pCR rate (24). A subsequent retrospective analysis of these two studies by this team found a high correlation between the dose of radiotherapy and the degree of tumor regression at doses within 50.4-70 Gy (25). They then performed a prospective trial with a high cCR rate of 78% after using the same treatment regimen as before (60 Gy/30F + 5 Gy brachytherapy with synchronized fluorouracil) and a 0% 2-year recurrence rate in patients who underwent surgery (26).

The RECTAL-BOOST trial found more patients with complete or near complete tumor regression (69.4% vs. 45.3%) in the intensive neoadjuvant radiotherapy group (15Gy/3F + 50Gy/25F synchronized capecitabine) compared to the standard treatment group (50Gy/25F synchronized capecitabine) despite not increasing pCR rates and sustained cCR rates (27). Moreover, the FDRT-002 trial and Bertocchi, Barugola (28) also showed that patients receiving enhanced radiotherapy failed to experience improved pCR rates and anal preservation rates but instead had prolonged wound healing and increased toxicity (29–31). Guido, Cuicchi (32) found that treating patients (n=18) with nCRT with a push to 50 Gy on top of 45 Gy/25F resulted in a pCR of 38.8%. A META analysis also found a slight increase in pCR rates after the radiotherapy dose was augmented with techniques such as nCRT+SIB or IMRT/VMAT (33).

The OPERA trial was a European phase 3 randomized trial in which group A patients (n=69) were treated with CRT (45 Gy/25/5 weeks), during which oral capecitabine was administered followed by enhanced external body radiation radiotherapy (EBRT) at 45 Gy/5 F/5 W, and group B patients (n=72) were treated with CRT followed by contact X-ray brachytherapy (CXB) (90 Gy/3 F/4 W). At week 24, cCR was observed in 64% and 92% of patients in groups A and B, respectively, with a final anal preservation rate of 59% in group A and 81% in group B. A total of 66/141 (47%) patients suspected of having residual tumor or local recurrence after obtaining cCR underwent surgery, and after more than 3 years of follow-up, the overall TME-free survival rates were 57% in group A and 79% in group B. Those who require surgery due to treatment failure can be salvaged with guaranteed radical treatment, and this study suggests that nonsurgical treatment with increased intensity of radiotherapy through intracavitary contact radiotherapy (Papillon radiotherapy) is feasible (34).

Combining the above experimental results, we found that increasing the dose of external irradiation radiotherapy may improve the tumor downgradation rate, but not necessarily result in pCR rate or survival benefit, and there is a possibility of increasing the incidence of adverse events, but the OPERA trial suggests that high-dose, low-fraction external irradiation therapy and intracavitary brachytherapy may provide more benefits. Therefore, the choice of radiation therapy dose and modality needs to be carefully considered.

### 2.3 Range of the target area

In neoadjuvant radiotherapy for patients with LARC, many studies have explored whether expanding the target area for patients with LARC could improve the clinical benefit as radiotherapy itself may cause toxic reactions and lead to a higher rate of surgical complications.

A prospective trial found a 5-year local control rate of 96.1%, 3- and 5-year OS of 89.4% and 87%, respectively, and reduced gastrointestinal toxicity when reducing the target area volume (containing only the primary focus, rectal mesentery, perirectal and presacral lymph nodes) in intermediate- and low-risk locally advanced rectal cancer (35). Song, Geng (36) found that excluding the ischiorectal fossa from the target area in nCRT did not affect tumor prognosis and reduced the incidence of surgical incisional complications (18). At present, when neoadjuvant radiotherapy is performed for LARC, the internal and external iliac vascular bifurcations are chosen as the upper boundary of the radiotherapy target area. The STELLAR trial attempted to lower the upper boundary of the neoadjuvant radiotherapy target area to the sacral promontory for low and locally advanced rectal cancers and observed improvements in the CR rate and R0 resection rate (37). For some distal rectal cancers that are at an earlier stage with no high-risk factors, clinicians can consider lowering the upper boundary of the radiotherapy target area to halfway below the sacrum to ensure for therapeutic efficacy and also minimize the adverse effects caused by radiotherapy. However, no large-scale clinical study exploring this issue in depth has been conducted in recent years. It is expected that other clinical trials will be performed in the future to gain further insights into this question. A retrospective analysis found a 0% 2-year lateral pelvic lymph node (LPLN) recurrence rate when IMRT-SIB was pushed to 56-60 Gy in patients with positive LPLNs and did not increase the incidence of radiotherapy-related toxicity or surgical complications (38). Chen, Liu (39) found that in LARC patients with isolated inguinal lymph node metastases, enhanced irradiation of the metastatic lymph nodes (IMRT-SIB 58 Gy/25 F) resulted in better LRR. The 3-year OS and local recurrence-free survival were 100% (39).

A study that included 399 patients found similar 5-year failure rates for EIN but increased 5-year failure rates for IN in patients with anal canal invasion compared to patients without anal canal invasion when irradiation of external iliac lymph nodes (EIN) and inguinal lymph nodes (IN) was not performed, although there was an increased 5-year failure rate for IN; however, there were no significant differences between the two groups in 5-year OS, DFS, distant metastasis-free survival (DMFS), or local recurrence-free survival (LRFS) (40). Another retrospective study included a total of 214 LARC patients with anal sphincter invasion but no ILN and ELN metastases and found that even without ILN and ELN irradiation, the 3-year failure rates for ILN and ELN were only 3.7% and 3.3%, respectively; however, tumor inferior margin invasion or location below the dentate line, high histologic grade, and perineural invasion were strongly associated with ILN and ELN treatment failure correlation (41). Therefore, in patients with LARC with low-risk factors, non-irradiation of ILNs and ELNs may be considered, thus reducing the incidence of adverse effects of radiotherapy without increasing the risk, but in high-risk patients, irradiation of ILNs and ELNs may still be needed.

RT plays an important role as a neoadjuvant treatment for patients with LARC; however, the PROSPECT trial revealed that not all patients with LARC seem to require RT. This trial included patients with cT2N+/cT3 rectal cancer, who were reclassified after chemotherapy and treated with surgery if the primary tumor regressed >20% or with preoperative RT if ≤20% regression was observed. The results showed that patients treated with neoadjuvant chemotherapy using the mFOLFOX6 regimen had similar 5-year DFS and LRR rates as the nCRT group, along with a significant decrease in toxicity. This study confirmed that neoadjuvant chemotherapy is effective in helping many patients avoid RT. Thus, it has great clinical value as a more cost-effective treatment for patients who cannot undergo RT, especially for young patients who have not yet had children, to reduce radiation-induced damage to the reproductive system (42). Additionally, for patients with cT3 low rectal cancer, the NAIR trial showed that neoadjuvant chemotherapy using only the mFOLFOX6/CAPOX regimen resulted in improved 3-year recurrence-free survival (75.5%) and prevented the effects of RT on patients’ surgery, postoperative wound healing and anal function (43).

## 3 Neoadjuvant chemoradiotherapy

The CAO/ARO/AIO-94, EORTC 22921 and FFCD 9203 trials made fluorouracil concurrent radiotherapy the standard neoadjuvant treatment regimen used to date. Although this treatment modality significantly improved the tumor stage reduction and pCR rate of patients compared with surgery alone or postoperative adjuvant radiotherapy modality, it did not significantly improve the DFS and OS of patients (2–5), so many studies explored other neoadjuvant treatment modalities.

In 2012, Hofheinz, Wenz (5) in Germany used capecitabine instead of fluorouracil for simultaneous radiotherapy after similar local control rates, lower distant metastasis rates, and even slightly higher 5-year OS and 3-year DFS. Based on this, nCRT with capecitabine has now also become one of the standard treatment options for LARC.

The addition of chemotherapeutic agents to nCRT to improve pCR rates has been explored by some authors, and the ACCORD 12 trial, which followed 598 patients for 3 years, found that although the addition of OX to capecitabine with concurrent radiotherapy did not increase the incidence of adverse events, it did not result in important benefits in terms of LRR, DFS, or OS (44). Several clinical trials in recent years have also found not only no improved clinical benefit but also increased toxicity with the addition of OX (45–49). In the FOWARC trial conducted at 15 centers in China, patients were equally assigned to the single-agent fluorouracil concurrent radiotherapy group, the mFOLFOX6 concurrent radiotherapy group and the mFOLFOX6 neoadjuvant chemotherapy group. Similar to previous findings, the mFOLFOX6 concurrent radiotherapy group did not outperform the single-agent fluorouracil concurrent radiotherapy group in terms of 3-year DFS and OS, but the mFOLFOX-RT group had the highest pCR rate (14% vs. 27.5% vs. 6.6%) and a similarly increased incidence of adverse events (50). The team published long-term follow-up results in 2023 and found that mFOLFOX6 plus radiotherapy also failed to improve long-term survival compared with fluorouracil plus radiotherapy and additionally did not provide long-term DFS (62.8% vs. 63%) and OS (73.2% vs. 73%) benefits to patients when compared with neoadjuvant chemotherapy with mFOLFOX6 alone (51). Although the addition of OX slightly improved the clinical benefit for patients, its increased toxic effects should not be underestimated; the disadvantages of this regimen may outweigh the benefits, and OX is not recommended as a routine neoadjuvant.

Many clinical studies have found the addition of irinotecan (CPT-11) to nCRT to have better efficacy and safety (52–58). The results of the CinClare study confirm the feasibility of the nCRT model of capecitabine in combination with CPT-11. The study enrolled 360 patients with wild-type or heterozygous mutations at the UGT1A1*28 locus, and a portion of the experimental group (CPT-11 combined with capecitabine concurrent chemoradiotherapy +1 cycle XELIRI) was treated with reduced doses due to the cumulative toxicity of the drug and radiotherapy, but the pCR rate in the experimental group was also 2-fold that of the control group (Capecitabine concurrent chemoradiotherapy +1 cycle XELOX). The pCR rate was as high as 40% in patients with sufficient doses of CPT-11 and four or more cycles, and the toxicity was within the tolerable range, providing good therapeutic prospects for patients with difficult R0 resection and a strong desire to preserve the anus (59).

## 4 Total neoadjuvant treatment

TNT is a more intense neoadjuvant treatment modality of SCRT followed by chemotherapy or induction/consolidation chemotherapy on top of nCRT that has become one of the standard treatment modalities for LARC (**Table 1**). However, individualized treatment regimens are needed for patients with LACR, thus avoiding overtreatment of patients or delaying the timing of surgery for patients with poor response (65, 80, 82).

**Table 1:** Clinical trials of total neoadjuvant therapy.

Study	Year(s) of Publication	Stage	Phase	n	Induction/ Consolidation chemotherapy	CRT Regime	pCR rate (%)	R0 Resection rate (%)	DFS(%)	OS(%)
STELLAR (37)	2022	cT3-4/N+	III	302	Con CAPOX(4 cycle)	25Gy/5F	17	92	3y-64.5	3y-87
				297		50Gy/25F+Cap	12	87.8	3y-62.3	3y-75
Chau, Brown (60)	2006	T4/T1-4N2	II	77	In CAPOX(4 cycles)	45Gy/25F+9Gy/5F+Cap	24	99	NR	NR
EXPERT Chua, Barbachano (61)	2010	T3-4/T1-4N2	II	105	In CAPOX(4 cycles)	54Gy+Cap	20	98	3y-68/5y-64	3y-83/5y-75
Schou, Larsen (62)	2012	T3-4/N+	NR	84	In CAPOX(2 cycles)	54Gy+Cap	23	94	3y-63.1/5y-63	3y-68.3/5y-67
GCR-3 Fernández-Martos, Pericay (63, 2015)	2010/2015	T3-4/N+	II	108	In CAPOX(4 cycles)	50.4Gy/28F+CAPOX	14	86	5y-62	5y-75
Maréchal, Vos (64)	2012	T3-4/T2N+	II	57	In mFOLFOX6(2 cycles)	45Gy/25F+5-FU	25	96	NR	NR
PRODIGE 23 Conroy, Bosset (65) and Etienne, Rio (66)	2021/2023	cT3-4	III	461	In FOLFIRINOX(6 cycles)	50Gy/25F+Cap/5-FU	28	95	3y-76/7y-68	3y-91/7y-82
Garcia-Aguilar, Chow (67) and Marco, Zhou (68)	2015/2018	T3-4N0/TxN1-2	II	259	Con mFOLFOX6(0/2/4/6 cycles)	45Gy/25F+5-FU	18/25/30/38	98/100/96/100	5y-50/81/86/76	5y-79/92/88/84
Zampino, Magni (69)	2009	T3-4/N+	II	51	Con Cap(2 cycles)	50.4Gy/28F+Cap	18	100	5y-85	NR
Zhu, Gu (70)	2013	T3-4/N+	II	42	Con Cap(1 cycle)	44Gy/20F+CAPOX	16	92	3y-57	3y-66
Gao, Zhang (71)	2014	T4/N+	II	36	Con CAPOX(1 cycle)	46-50Gy/23-25F+CAPOX	36	100	NR	NR
Gao, Lin (72)	2014	T3-4/N+	II	51	In+Con CAPOX(1 cycle)	50Gy/25F+CAPOX	42	100	NR	NR
CAO/ARO/AIO-12 Fokas, Allgäuer (73, 2022)	2019/2022	cT3-4/N+	II	306	In/Con 5-FU+LV+OX(3 cycles)	50.4Gy/28F+5-FU+OX	In 17/Con 25	In 92/Con 90	3y-In 73/Con 73	3y-In 92/Con 92
KCSG CO 14-03 Kim, Joo (74)	2018	cT3-4	II	108	Con CAPOX(2 cycle)	50.4Gy/28F+Cap	13.6	87	NR	NR
Myerson, Tan (75) and Markovina, Youssef (76)	2014/2017	cT3-4	II	76	Con mFOLFOX6(6 cycles)	25Gy/5F	28	71	3y-85	3y-96
Polish Bujko, Wyrwicz (77) and Ciseł, Pietrzak (78)	2016/2019	cT3-4	III	261	Con FOLFOX4(3 cycles)	25Gy/5F	16	77	3y-53/8y-43	3y-73/8y-49
				254		50.4Gy/28F+5-FU+OX	12	71	3y-52/8y-41	3y-65/8y-49
Chakrabarti, Rajan (79)	2021	cT3-4/N+	single-arm	69	Con CAPOX(2 cycle)	25Gy/5F	12	87	NR	NR
				71		50.4Gy/28F+Cap	10	90	NR	NR
RAPIDO Bahadoer, Dijkstra (80) and Dijkstra, Nilsson (81)	2021/2023	cT4a-bN2	III	460	ConCAPOX(6 cycle)/FOLFOX4(9 cycle)	25Gy/5F	28	91	3y-24	3y-89
				446		(50-50.4Gy/25-28F)+Cap	14	91	3y-30	3y-89

n, number of patients; RT, radiotherapy; CRT, chemoradiotherapy; SCRT, short course radiation therapy; pCR, pathologic complete response; R0, microscopically clear resection; DFS, disease-free survival; OS, overall survival; 5-FU, 5-fluorouracil; Cap, capecitabine; In, induction chemotherapy; Con, consolidation chemotherapy; CAPOX, capecitabine/oxaliplatin; FOLFOX, 5-fluorouracil, leucovorin, and oxaliplatin; Gy, gray; mFOLFOX6, modified FOLFOX6; FOLFIRINOX, 5-fluorouracil, leucovorin, irinotecan and oxaliplatin; -y, -year; NR, not reported.

### 4.1 Induction chemotherapy-nCRT

Chau, Allen (83) found in 2003 that the use of mitomycin before nCRT improved nCRT efficacy, after which they first tried the effect of the induction chemotherapy TNT treatment modality in patients with LARC; they found a high tumor efficiency of 97% and a pCR rate of 24% after 4 cycles of CAPOX induction chemotherapy before nCRT, and 48% of patients were close to CR (60). Subsequent EXPERT studies also found high pCR rates (20%) and R0 resection rates (96%) after induction chemotherapy using 3-4 cycles of CAPOX regimens and better 3-year relapse-free survival (RFS), DFS and OS (74%, 68%, 83%) with tolerable toxicity (61). Several other studies have also confirmed that the TNT treatment modality of induction chemotherapy does improve clinical benefit (62, 63, 2015).

Maréchal, Vos (64) found no clinical benefit after induction chemotherapy with 2 cycles of mFOLFOX6, but Cercek, Goodman (84) found a CR rate of 36% (pCR 21.3%, cCR 14.7%) in a retrospective analysis of 61 patients who underwent nCRT after induction chemotherapy with the mFOLFOX6 regimen. The overall CR rate was even higher in patients who received 8 cycles of induction chemotherapy at 40% (pCR 29%, cCR 11%), and all patients had varying degrees of tumor regression, a difference that may be related to induction chemotherapy for more cycles. In addition, 65% of lymph node-positive patients reached ypN0 after TNT (84), and in another retrospective study, the incidence of ypN0 was similarly found to be significantly higher after induction chemotherapy than in the nCRT group (75% vs. 62%) (85). Kim, Marco (86) also found that even though patients treated with TNT had a more advanced tumor stage, they still outperformed the nCRT group in terms of CR rate.

The PRODIGE 23 trial in France used a high-intensity mFOLFIRINOX regimen for induction chemotherapy, and although the 3-year OS rates were similar in the 2 groups, the 3-year DFS (76% vs. 69%), distant metastasis (17% vs. 25%), and pCR rates (28% vs. 12%) were significantly better in the induction chemotherapy group (n=231) than in the control group, which used nCRT (n=230), and the incidence of grade ≥3 adverse events was lower (65). After 7 years of follow-up, 42 patients died in the trial group compared to 55 in the control group, and all survival endpoints, such as OS, DFS, and MFS, were better in the trial group than in the control group; the 7-year LRR was also better in the trial group than in the control group (66). Compared to nCRT, induction chemotherapy provides not only improved clinical benefit but also greater flexibility. Due to the heterogeneity of LARC, however, a comprehensive clinical assessment of treatment benefit is still needed for patients.

### 4.2 nCRT-consolidation chemotherapy

Because different patients have different sensitivities to nCRT and are at risk of tumor progression or metastasis in the interval between surgeries, many studies have explored whether the addition of systemic therapy in the interval between surgeries can reduce the risk of tumor progression, promote tumor regression, or increase the pCR rate (67, 68, 74, 87, 88).

Habr-Gama, Perez (87) found that continuation of fluorouracil chemotherapy after nCRT improved CR rates. A prospective phase II trial conducted at 17 centers in the United States and Canada found that consolidation chemotherapy with the mFOLFOX6 regimen improved pCR rates in patients and that patients with 4 and 6 cycles had higher pCR rates than those with only 2 cycles of consolidation chemotherapy. In addition, the incidence of grade ≥3 adverse events decreased instead with increasing cycles of consolidation therapy, and the use of consolidation chemotherapy was also found to improve DFS after 5 years of follow-up (67, 68).

After Zampino, Magni (69) found that continuation of capecitabine after nCRT improved clinical benefit, Zhu, Gu (70) also found that consolidation chemotherapy with capecitabine after nCRT with OX+capecitabine achieved better tumor step-down rates. A subsequent prospective trial by Gao, Zhang (71) found that nCRT with the XELOX regimen followed by one cycle of chemotherapy with the XELOX regimen significantly improved the pCR rate (36.1%) and that 44.4% of patients approached pCR with good tolerability (71). This finding was confirmed in several other clinical trials (74, 88). Gao, Lin (72) in 2014 found that “sandwich” neoadjuvant therapy (XELOX+nCRT+XELOX) resulted in pCR in 42.2% (19/45) of patients, not including 18 patients (40%) who approached pCR. In addition, 18 patients (40%) achieved pCR. Meanwhile, the toxic reaction is within the control range.

The CAO/ARO/AIO-12 study by Fokas, Allgäuer (73, 2022) found higher patient compliance and pCR rates with consolidation chemotherapy TNT than in patients treated with induction chemotherapy TNT (91% vs. 97% and 17% vs. 25%) and a lower incidence of grade 3/4 adverse events (37% vs. 27%), but both groups had the same 3-year PFS of 73% and had similar 3-year LRR (6% vs. 5%); however, the incidence of grade 3/4 chronic toxicity was slightly lower in the consolidation chemotherapy group than in the induction chemotherapy group (11.8% vs. 9.9%). Although this study concluded that consolidation chemotherapy regimens could be the preferred TNT sequence, tumor response is influenced by a variety of factors, and in clinical practice it is still necessary to develop an appropriate treatment regimen after a thorough patient assessment to provide maximum clinical benefit to patients.

### 4.3 Short course of radiotherapy - consolidation chemotherapy

Although earlier studies found better local control with nCRT than with surgery immediately after SCRT (19, 2006, 21), the Stockholm III Trial and several other studies found that delaying surgery after SCRT also resulted in better tumor downstaging (89, 90, 2015, 91). Widder, Herbst (92) found that chemotherapy administered in the interval between surgeries may improve outcomes.

Myerson, Tan (75) found that consolidation chemotherapy using a 4-cycle mFOLFOX6 regimen after SCRT (25 Gy/5 F) led to a 28% total CR and had lower toxicity and better 3-year DFS (85% vs 68%) (76). The published results of the 2016 Polish trial indicated that SCRT followed by 3 cycles of FOLFOX4 produced better R0 resection (77% vs. 71%) and pCR rates (16% vs. 12%) than nCRT and was even not inferior to nCRT in terms of 3-year DFS (53% vs. 52%) and OS (73% vs. 65%) (77); after long-term follow-up, however, there was no significant difference in survival benefit between the two groups (78). Myerson, Tan (75) then performed a matched pair analysis between their trial and the Polish trial and found that SCRT + consolidation chemotherapy had better pCR rates (28% vs. 16%), tumor step-down rates (75% vs. 41%), 3-year DFS (85% vs. 68%) and 3-year OS (96% vs. 88%) in terms of gains, and TNT treatment was found to be significantly associated with a lower risk of recurrence by subgroup analysis. These studies provided the rationale for SCRT + consolidation chemotherapy to later become the standard neoadjuvant treatment modality for LARC.

Chakrabarti, Rajan (79) compared the efficacy of SCRT + consolidation chemotherapy (5 x 5 Gy sequential 2 cycles of XELOX) or nCRT (50 Gy/25 F simultaneous capecitabine) followed by sequential 6 cycles of XELOX regimen, and patients were found to have better compliance with SCRT+ consolidation chemotherapy (63% vs. 41%) and shorter treatment duration, higher tumor step-down rates, lower incidence of grade 3/4 acute toxicity (2% vs. 4%), and similar to nCRT+ consolidation chemotherapy in terms of R0 resection rate (87% vs. 90%), pCR rate (12% vs. 10%), and overall tumor step-down (75% vs. 75%). The RAPIDO trial found a significantly lower 3-year disease-related treatment failure rate (23.7% vs. 30.4%) in patients in the experimental group (5 × 5 Gy followed by sequential 6 cycles of CAPOX/4 cycles of FOLFOX4 consolidation chemotherapy, n=462) compared to patients in the standard treatment group (50.4Gy/28F or 50Gy/25F with concurrent capecitabine, n=450) (80); the 5-year local area failure (12% vs. 8%) and local recurrence rates (10% vs. 6%) were higher than in the standard treatment group, and the experimental group was associated with an increased risk of local recurrence, but the rates of disease-related treatment failure and distant metastases remained better in the experimental group than in the standard treatment group (81). The STELLAR trial conducted at 16 centers in China compared the efficacy of preoperative SCRT + consolidation chemotherapy (5 × 5 Gy followed by sequential 4 cycles of CAPOX) with standard nCRT (50 Gy/25 F synchronized capecitabine) and found that the overall CR rate (21.8% vs. 12.3%) and R0 resection rate (91.5% vs. 87.8%) in patients treated with TNT were higher than those in the CRT group, and the 3-year DFS (64.5% vs. 62.3%) and MFS (77.1% vs. 75.3%) were significantly better than those in the CRT group (86.5% vs. 75.1%), while the 3-year LRR rate was lower in the TNT group (8.4% vs. 11%) (37). However, Romesser, Park (93) of Memorial Sloan Kettering Cancer Center presented the results of their trial at ASCO 2023; their trial involved assigning 332 patients treated with TNT to the LCRT group (n = 256) and the SCRT group (n = 76). Patients in both groups had a cCR of 46%, but overall, patients who had radiotherapy first had a higher cCR than patients who had chemotherapy first [53% vs. 44% (LCRT), 52% vs. 43% (SCRT)], and then after more than 2 years of follow-up, the LCRT group outperformed the SCRT group in terms of organ preservation (40% vs. 29%) but not in terms of 2-year OS (95% vs. 92%), DFS (78% vs. 70%), distant recurrence rate (20% vs. 21%) and watchful waiting rate (98% vs. 94%) were similar between the two groups, but it should be noted that among patients who opted for W&W treatment, the 2-year local regeneration rate was 20% in the LCRT group compared to 36% in the SCRT group (93). The ongoing ACO/ARO/AIO-18.1 trial may further validate whether the TNT treatment modality with LCRT results in a higher organ preservation rate. Although many studies have concluded that the short-term benefit of SCRT + consolidation treatment modality is not inferior or even superior to nCRT, more clinical studies are needed to validate it in terms of long-term survival benefit. Both nCRT and short-course radiotherapy have now become standard neoadjuvant treatment modalities. Despite the increased acute toxicity of SCRT, clinical benefit can be achieved with shorter treatment duration, lower economic cost, more flexible treatment modalities and better compliance than nCRT.

## 5 Combination with immunotherapy

Immunologic agents (ICIs) have shown good efficacy in advanced rectal cancer patients with dMMR/MSI-H (94, 95), and the application of ICIs in rectal cancer is very limited because the majority of rectal cancer patients are of the MSS type. Some studies have demonstrated that radiotherapy can enhance the antitumor immune response by upregulating PD-L1 expression, altering the tumor microenvironment, inducing immunogenic death of tumor cells (ICD), etc., to enhance the antitumor immune response of patients (96–99), so many studies in recent years have explored whether the addition of immune drugs to neoadjuvant therapy for LARC can provide better benefit (**Table 2**).

**Table 2:** Clinical trials of neoadjuvant chemoradiotherapy combined with the immunotherapy.

Study	Year(s) of Publication	Stage	Phase	n	Pathology	Study design	CRT Regime	pCR rate (%)	DFS(%)	OS(%)	Other Results
Voltage-A Bando, Tsukada (100) and Tsukada, Bando (101)	2022	T3-4N0-2	II	39	MSS	CRT-nivolumab(4 cycles)	50.4Gy/28F+Cap	30	NR	3y-97	NR
NSABP FR-2 George, Yothers (102)	2022	stage II/III	II	45	MSS	CRT-durvalumab(4 cycles)	NR	22	NR	NR	R0(%) 81%
PANDORA Tamberi, Grassi (103)	2022	cT3-4N+	II	55	NR	CRT-durvalumab(3 cycles)	50.4Gy/28F+Cap	32.7	NR	NR	NR
AVANA Bensi, Salvatore (104)	2021	cT3-4/N+	II	101	NR	CRT+avelumab(6 cycles)	50.4Gy/28F+Cap	23	NR	NR	MPR (%) 62
R-IMMUNE Carrasco, Schröder (105)	2021	stage II/III	Ib/II	25	NR	CRT+atezolizumab(4 cycles)	50Gy/25F+5-FU	24	NR	NR	NR
NRG-GI002 Rahma, Yothers (106) and George, Yothers (107)	2021	cT3-4	II	90	MSS	FOLFOX(8 cycles)-CRT+pembrolizumab(6 cycles)	50.4Gy/28F+Cap	32	3y-64	3y-95	NR
				95	MSS	FOLFOX(8 cycles)-CRT	50.4Gy/28F+Cap	29	3y-64	3y-87	NR
Zhou, Yu (108)	2022	T1-3aN1-2	II	23	MSS	[RT+sintilimab(2 cycles)]-[Cap/CAPOX(6 cycles)+sintilimab(2 cycles)]	50Gy/25F	9	NR	NR	MPR (%) 60
PKUCH 04 Wu, Li (109)	2022	NR	II	25	MSS	[CAPOX+Camrelizumab](3 cycles)-CRT-CAPOX(2 cycles)	50.4Gy/28F+Cap	33	NR	NR	MPR (%) 90
Yao, Yang (110)	2022	cT3N0/cT1-3N1-2	II	20	pMMR	[CRT+tislelizumab(2 cycles)]-[Cap+sintilimab](1 cycles)	50Gy/25F+Cap	58	NR	NR	NR
Lin, Cai (111)	2021	T3-4N0/T1-4N+	II	30	pMMR+dMMR	SCRT-[CAPOX+camrelizumab](2 cycles)	25Gy/5F	48	NR	NR	NR
Shamseddine, Zeidan (112)	2020	stage II/III	II	44	pMMR	SCRT-[mFOLFOX6+avelumab](6 cycles)	25Gy/5F	38	NR	NR	MPR (%) 68

n, number of patients; RT, radiotherapy; CRT, chemoradiotherapy; SCRT, short course radiation therapy; pCR, pathologic complete response; R0, microscopically clear resection; DFS, disease-free survival; OS, overall survival; MPR,; major pathological response 5-FU, 5-fluorouracil; Cap, capecitabine; In, induction chemotherapy; CAPOX, capecitabine/oxaliplatin; FOLFOX, 5-fluorouracil, leucovorin, and oxaliplatin; Gy, gray; mFOLFOX6, modified FOLFOX6; -y, -year; NR, not reported.

In the NICHE study, among patients with locally advanced mismatch repair-deficient colon cancer, 95% achieved an MPR, and 60% had pCR after treatment with ipilimumab + nivolumab (113). In the PICC study, patients with locally advanced mismatch repair-deficient colon cancer achieved a high pCR rate after 6 cycles of toripalimab neoadjuvant therapy (114). Considering the remarkable efficacy of immunotherapy in colorectal cancer, at the 2022 ASCO Annual Meeting, Cercek et al. demonstrated that a 100% cCR rate could be achieved with dostarlimab alone for patients with dMMR/MSI-H (115). Additionally, Yang et al. used different types of PD-1 inhibitors, including pembrolizumab, sintilimab, and tislelizumab, to treat 20 LARC patients with dMMR/MSI-H. As a result, 90% of the patients experienced CR, of whom 11 achieved pCR postoperatively, and the remaining 7 had cCR or near-cCR, with 2-year DFS and OS rates of 100% (116). Further, Chen et al. found that 12 out of the 16 patients in their study achieved CR, further demonstrating the significant efficacy of immuno-monotherapy as a neoadjuvant treatment for LARC patients with dMMR/MSI-H (117). However, more than 90% of patients with colon and rectal cancers have MSS tumors, and these patients are usually not sensitive to immunotherapy. Radiotherapy has been demonstrated to increase the efficacy of immunotherapy. Thus, many studies have attempted to use immunotherapy in combination with radiotherapy to treat LARC patients with MSS tumors and have achieved good results.

The VOLTAGE-A trial was the first to attempt nCRT in combination with ICI as neoadjuvant therapy, and with the use of 5 cycles of nivolumab after nCRT, 30% of 37 LARC patients with MSS achieved pCR with a major pathologic response rate of 38%, and 6 of 8 patients with PD-L1 expression ≥1% achieved pCR. In tumors, the pCR rate was 78% in 9 patients with a CD8+ T-cell/effector regulatory T-cell (CD8/eTreg) ratio ≥2.5 in infiltrating lymphocytes (100), and in patients with MSS, 3-year RFS and OS reached 79.5% and 97.4%, respectively, while patients with MSI-H had 100% for both metrics (101). Despite the small sample size of this study, the combination of nCRT with ICI can be considered a very promising neoadjuvant treatment option for application. In addition, the NSABP FR-2 trial in the United States and the PANDORA trial in Italy found that sequential administration of durvalumab after nCRT also resulted in better pCR rates (22.2% and 32.7%), and an additional 25.5% of patients in the PANDORA trial approached pCR and had higher rates of tumor stage reduction and anal preservation (102, 103).

The 2021 AVANA trial found that 23% of patients achieved pCR and 61.5% achieved major pathologic response (MPR) after 6 cycles of avelumab (10 mg/kg/2 weeks) synchronized with Ncrt (104). The R-IMMUNE trial included 25 patients with 4 cycles of atezolizumab synchronized with nCRT, of whom 6 (24%) patients achieved pCR and only 13% had ≥ grade 3 or higher adverse reactions (105). Although these 2 trials did not result in stunning efficacy, this presents a new treatment option for patients who are not sensitive to chemotherapy.

The NRG-GI002 trial explored the impact of adding pembrolizumab after induction chemotherapy TNT treatment and found that the use of pembrolizumab did not increase pCR rates (29.4% vs. 31.9%) and cCR rates (13.6% vs. 13.9%) but increased the incidence of grade ≥3 or higher adverse reactions (48.2% vs. 37.3%) (106); after 3 years of follow-up, however, the addition of pembrolizumab was found to significantly increase the patients’ 3-year OS improvement (87% vs. 95%) (107). Thus, sequential use of ICIs after more intense TNT may have a long-term survival benefit but does not further increase short-term efficacy; instead, it leads to greater toxicity in patients due to overtreatment. A single-arm, prospective phase II trial conducted in China explored the efficacy and safety of sintillimab in ultralow MSS/pMMR in patients with locally advanced rectal cancer. The trial enrolled 23 patients who received nCRT synchronized with 2 cycles of sintillimab followed by 6 cycles of capecitabine or CAPOX regimen plus 2 cycles of sintillimab as consolidation therapy. Of the 10 patients who underwent the surgery, 2 patients achieved pCR, with an overall CR rate (cCR+pCR) of 52.2% and an anal preservation rate of 95.5% (108). And another phase II study in China found that adding Tislelizumab to TNT treatment surprisingly increased the pCR rate to 58% (110). In contrast, the PKUCH-04 study used a more intense sandwich neoadjuvant treatment combined with ICI model, with simultaneous camerelizumab during 3 cycles of CAPOX induction chemotherapy followed by nCRT and 2 cycles of CAPEOX consolidation chemotherapy; seven (33.3%) of the 25 patients with MSS achieved pCR and 4 (16%) patients achieved cCR, while grade 3 adverse events were mainly lymphopenia (24%) and no grade 4 adverse events occurred (109). Although the PKUCH04 study achieved better trial results than the NRG-GI002 trial, this outcome cannot be ruled out as being due to intensive chemotherapy. Furthermore, lymphocytes are very sensitive to radiation, whereas the NRG-GI002 trial using pembrolizumab after nCRT likely decreased the patient’s antitumor immune response. Therefore, in addition to focusing on the impact of treatment intensity on the patient, it is also important to consider whether the different treatment sequences will differ in efficacy.

Many studies in recent years have found that the use of SCRT + consolidation chemotherapy modality has noninferior efficacy to nCRT (37, 75, 80, 96–103). Lin, Cai (111) first combined SCRT with ICI (25 Gy/5F + 2 cycles of CAPOX synchronized with camrelizumab), and 26 of the 27 patients they included were pMMR. At the end of neoadjuvant therapy, 13 patients (48.1%) achieved pCR, and all patients achieved R0 resection, with an 88.9% anus preservation rate and no grade ≥4 adverse events (111). The efficacy of adding toripalimab to TNT treatment was explored in the TORCH trial, which was designed with an induction chemotherapy arm (2 cycles of CAPOX combined with toripalimab + SCRT) and 2 treatment regimens in the consolidation chemotherapy group (SCRT + 6 cycles of CAPOX combined with toripalimab). Of the 48 patients who completed treatment, 50.0% achieved cCR, with an overall CR rate of 60.4% (29/48), a pCR rate of 60.7% (17/28), an MPR rate of 78.6% (22/28), and an anal preservation rate of 88.9% (40/45) (118).The Averectal trial found that with 6 cycles of mFOLFOX6 combined with avelumab after SCRT (25 Gy/5 F), 3 of 12 patients with pMMR (25%) achieved pCR, 3 more patients (25%) approached pCR, 50% had a major pathologic response, and no grade 4 or higher adverse events occurred (112). Dai, Wang (119) designed an open-label, single-center, phase II study to explore the efficacy and safety of using envafolimab plus CAPEOX after SCRT in patients with MSS/pMMR locally advanced rectal cancer, and 8 of 12 patients (76.6%) who have completed neoadjuvant therapy thus far have achieved pCR; their final trial results are expected to be outstanding. Compared to the previously mentioned treatment modality of nCRT+ICI, SCRT+ICI showed important advantages, which may be because the high-dose, low-fractionation radiotherapy modality has less impact on lymphocytes and may be because high-dose radiotherapy can increase the patient’s antitumor immune response. In conclusion, SCRT+ICI may be a more favorable neoadjuvant immunotherapy regimen for patients.

## 6 Combination with targeted agents

Targeted agents notably improve the survival benefit of patients with metastatic rectal cancer, and targeted agents can exert antitumor effects by inducing apoptosis and inhibiting angiogenesis (6). By combining targeted agents with radiotherapy, the action properties of targeted agents may be better exploited (120, 121), so there are many studies exploring neoadjuvant therapy combined with targeted agents (**Table 3**).

**Table 3:** Clinical trials of neoadjuvant chemoradiotherapy combined with targeted agents.

Study	Year(s) of Publication	Stage	Phase	n	Study design	CRT Regime	pCR rate (%)	R0 Resection rate (%)	DFS(%)	OS(%)	LRR
Willett, Duda (122, 2010)	2009/2010	T3-4	II	32	CRT+Bev(4 cycles)	50.4Gy/28F+5-FU	16	NR	5y- 67	5y- 95	5y- 0%
Crane, Eng (123)	2010	cT3N0-1	II	25	CRT+Bev(3 cycles)	50.4Gy/28F+Cap	32	NR	2y- 77	2y-100	2y- 4%
Yu, Wang (124)	2018	stage II/III	II	45	[CAPOX+Bev](1 cycle)-[CRT+Bev(2 cycles)]	50Gy/25F+CAPOX	40	100%	3yPFS- 89	3y- 95	3y- 0%
Velenik, Ocvirk (125)	2011	T2-4N0-2	II	61	Bev(1 cycle)-CRT+Bev(3 cycles)	50.4Gy/28F+Cap	13	NR	NR	NR	NR
Gasparini, Torino (126)	2012	T3-4/T2N+	II	43	CRT+Bev(4 cycles)	50.4Gy/28F+Cap	14	95%	3y- 75	NR	3y- 12%
ECOG 3204 Landry, Feng (127)	2013	T3-4	II	54	CRT+Bev(3 cycles)	50.4Gy/28F+CAPOX	17	88%	NR	NR	NR
Dellas, Höhler (128)	2013	cT3-4N+	II	70	CRT+Bev(3 cycles)	50.4Gy/28F+CAPOX	17	94%	NR	NR	NR
INOVA Borg, André (129, 2019)	2014/2019	T3	II	46	[FOLFOX4+Bev](4 cycles)-[CRT+Bev(3 cycles)]	45Gy/25F+5-FU	24	98%	5y- 70	5y- 91	5y- 7%
				45	CRT+Bev(3 cycles)	45Gy/25F+5-FU	11	98%	5y- 64	5y- 73	5y- 9%
TRUST Masi, Vivaldi (130)	2019	cT3-4/N+	II	48	[FOLFOXIRI+Bev](6 cycles)-[CRT+Bev(3 cycles)]	50.4Gy/28F+Cap	36	NR	2y- 81	NR	3y- 8%
Marti, Jayson (131)	2019	T3	I	18	CRT+Cediranib	45Gy/25F+Cap	12	100%	NR	NR	NR
Bertolini, Chiara (132)	2009	cT3-4N0-1	II	40	Cet(3 cycles)-[CRT+Cet(5 cycles)]	50–50.4 Gy/25-28F+5-FU	8	NR	NR	NR	NR
MARGIT Horisberger, Treschl (133)	2009	cT3-4/N+	II	50	CRT+Cet(5 cycles)	50.4Gy/28F+CAPIRI	8	100%	NR	NR	NR
Kim, Hong (134)	2011	cT3-4/N+	II	39	CRT+Cet(6 cycles)	50.4Gy/28F+Cap	23	100%	3y- 80	3y- 94	NR
EXPERT-C Dewdney, Cunningham (135)	2012	T3-4	II	165	[CAPOX+Cet](4 cycles)-[CRT+Cet]	50.4Gy/28F+Cap	15	96%	NR	NR	NR
Valentini, De Paoli (136)	2008	T3/T2N+	I/II	41	CRT+Gef	50.4Gy/28F+5-FU	27	97%	NR	NR	NR
StarPan/STAR-02 Pinto, Di Fabio (137)	2011	T3N+/T4	II	60	Pan(1 cycle)-[CRT+Pan(2 cycles)]	50.4Gy/28F+FOLFOX	21	78%	NR	NR	NR
RaP Study/STAR-03 Pinto, Di Bisceglie (138)	2018	cT3/T2-3N+	II	98	RT+Pan(3 cycles)	50.4Gy/28F	11	91%	NR	NR	NR
SAKK 41/07 Helbling, Bodoky (139)	2013	T3-4/N+	II	40	CRT+Pan(4 cycles)	45Gy/25F+Cap	10	85%	NR	NR	NR

n, number of patients; RT, radiotherapy; CRT, chemoradiotherapy; pCR, pathologic complete response; R0, microscopically clear resection; DFS, disease-free survival; OS, overall survival; LRR, local recurrence rate; 5-FU, 5-fluorouracil; Cap, capecitabine; In, induction chemotherapy; Bev, bevacizuma; Cet, cetuximab; Gef, Gefitinib; Pan, panitumumab; CAPOX, capecitabine/oxaliplatin; FOLFOX, 5-fluorouracil, leucovorin, and oxaliplatin; Gy, gray; mFOLFOX6, modified FOLFOX6; FOLFOXIRI, 5-fluorouracil, leucovorin, irinotecan and oxaliplatin; -y, -year; NR, not reported.

Bevacizumab is a VEGF inhibitor (140). nCRT was first combined with bevacizumab by Willett, Duda (122), and despite only 15.6% of patients achieving pCR, the 5-year OS was 95%, DFS and distant metastasis rate was 68.9%, local control rate was 91.7%, and no serious toxicity occurred (141). However, Crane, Eng (123) found that after using bevacizumab every 2 weeks during nCRT, 8 (32%) patients achieved pCR and 6 (24%) patients approached pCR, with 2-year local recurrence in 1 patient, 2-year distant metastases in 3 patients, and 2-year DFS and OS rates of 77.3% and 100%, respectively. While the addition of bevacizumab during nCRT was also found in several other studies to increase the rate of anal preservation, pCR rate and survival benefit to varying degrees (124, 142–144), Velenik, Ocvirk (125) found that combining bevacizumab during nCRT did not improve the pCR rate (13.3%), although it did not increase toxicity. Several other studies also found that nCRT combined with bevacizumab did not improve pCR, distal recurrence-free survival, DFS or 5-year OS (126, 145, 146). In addition, Dipetrillo, Pricolo (147) found that the combination of bevacizumab during TNT not only did not improve the pCR rate (20%) but also produced very high toxicity, with grade 3/4 toxicity, mainly gastrointestinal and hematological, in 19 of 25 patients (76%), which led to the early termination of the trial; the same was found in 2 other studies (127, 128). This may be due to the use of OX-containing chemotherapy regimens, but even with the less intense neoadjuvant regimen (45 Gy/25F + capecitabine 825 mg/m2 bid + bevacizumab 5 mg/kg, d1, d15 and d29), more than 50% of patients still experienced ≥ grade 3 adverse effects (148). The INOVA trial explored the impact of radiotherapy in neoadjuvant targeted therapy for LARC; the researchers enrolled 91 patients, assigned them in a 1:1 ratio and found that the use of bevacizumab in combination with 5-FU-RT significantly improved pCR rates (23.8% vs. 11.4%) and 5-year OS (90.5% vs. 72.7%) compared with bevacizumab in combination with FOLFOX4 and had similar rates of adverse events (129, 2019). The TRUST study included 48 patients with LARC who received 6 cycles of FOLFOXIRI-synchronized bevacizumab induction therapy followed by nCRT (50.4 Gy synchronized capecitabine) in combination with bevacizumab and found a 2-year DFS of 80.45% and a pCR in 36.4% of the 44 patients who underwent surgical resection (130). The NOMINATE trial is exploring the impact of adding bevacizumab to TNT therapy, but the results have not yet been reported, so we look forward to their results (149). The DREAM therapy trial found that starting another panvascular endothelial growth factor (pan-VEGF) receptor tyrosine kinase inhibitor, cediranib, 10 days before nCRT and continuing it until the end of nCRT significantly increased the excellent clinical or pathological response rate (41%) (131). Although the use of bevacizumab during neoadjuvant therapy may not improve the benefit, it certainly increases toxicity and therefore is not recommended for patients during nCRT, but cediranib seems to have the potential to provide a higher benefit for patients. Although the addition of bevacizumab to neoadjuvant therapy was not successful, Yara L identified a new treatment regimen in patients with clinical stage ≤T3ab N0-1 distal-mid rectal tumors without mesorectal fascia involvement using SCRT followed by sequential 3 cycles of atezolizumab + bevacizumab that achieved cCR or near cCR in 56% of patients; although this trial has not yet been completed, it offers good prospects for the use of bevacizumab in neoadjuvant therapy for LARC (150).

The combination of EGFR inhibitors with RT has a synergistic effect because RT increases EGFR expression in tumor cells, and blocking EGFR can make cells more sensitive to radiotherapy (149, 150). Ten years ago, a study confirmed the high safety profile of adding cetuximab to neoadjuvant therapy for LARC; however, regardless of the neoadjuvant regimen used, after adding cetuximab, pCR rates were disappointingly low (150–154). Although some studies have also demonstrated better pathological step-down rates and survival benefit in patients after the addition of cetuximab (155–157), they were not significantly better than nCRT or TNT, so the addition of cetuximab to nCRT is not recommended. In contrast, the addition of gefitinib, also an EGFR inhibitor, to nCRT increased the pCR rate to 30.3%, but unfortunately, this also significantly increased gastrointestinal toxicity in patients (158). The StarPan/STAR-02 trial tried another EGFR inhibitor, panitumumab, and found that the addition of fluorouracil + oxaliplatin with concurrent radiotherapy with panitumumab resulted in a pCR rate of 21.1%, again with improved gastrointestinal toxicity (159). Although this regimen did not significantly improve the pCR rate in patients, it seemed to suggest the potential for panitumumab to provide benefit to LARC patients, and on this basis, the RaP Study/STAR-03 study attempted to combine FOLFOX4 synchronous radiotherapy with panitumumab in KRAS wild-type LARC patients; disappointingly, this regimen did not increase the pCR rate in patients (10.9%) (160). The SAKK 41/07 trial attempted to add panitumumab during nCRT in KRAS wild-type LARC patients, and interestingly, although the addition of panitumumab did not increase the pCR rate in patients (10% vs. 18%), it did increase the proportion of patients approaching pCR (43% vs. 14%) (161). Overall, the addition of EGFR inhibitors does not appear to provide additional benefit to LARC patients but rather has the potential to cause additional gastrointestinal toxicity. Therefore, the addition of EGFR inhibitors during neoadjuvant therapy in LARC patients is not recommended.

## 7 Summary

In the past two decades, the neoadjuvant treatment methods and tools for locally advanced rectal cancer have continued to advance, not only achieving better and better results in terms of efficacy but also decreasing toxicity. TNT therapy has now surpassed nCRT as the most common neoadjuvant treatment option for clinical LARC, greatly reducing the difficulty of TME surgery and promoting a better prognosis for patients. Although targeted drugs have not demonstrated their efficacy in neoadjuvant therapy for LARC, the promising therapeutic prospects shown by immunologic drugs provide clinicians and patients with another new option. Due to the differences in genotype, tumor location and size, extent of tumor invasion, and patient’s physical condition, a multidisciplinary discussion (MDT) is still needed to carefully assess individual situations and develop the most appropriate treatment plan for each patient.

## Author contributions

ZY: Data curation, Formal Analysis, Funding acquisition, Investigation, Methodology, Project administration, Software, Visualization, Writing – original draft, Writing – review & editing. YYH: Data curation, Formal Analysis, Writing – original draft. YHH: Formal Analysis, Software, Writing – original draft. LL: Methodology, Writing – original draft. XH: Conceptualization, Project administration, Resources, Writing – original draft, Writing – review & editing. SQ: Writing – original draft, Writing – review & editing, Data curation, Formal Analysis, Investigation, Methodology, Project administration, Software, Supervision.

## References

[1] Siegel RL Miller KD Fuchs HE Jemal A. Cancer statistics, 2022. CA Cancer J Clin (2022) 72(1):7–33. doi: 10.3322/caac.21708 35020204

[2] Sauer R Liersch T Merkel S Fietkau R Hohenberger W Hess C. Preoperative versus postoperative chemoradiotherapy for locally advanced rectal cancer: results of the German CAO/ARO/AIO-94 randomized phase III trial after a median follow-up of 11 years. J Clin Oncol (2012) 30(16):1926–33. doi: 10.1200/JCO.2011.40.1836 22529255

[3] Gérard JP Conroy T Bonnetain F Bouché O Chapet O Closon-Dejardin MT. Preoperative radiotherapy with or without concurrent fluorouracil and leucovorin in T3-4 rectal cancers: results of FFCD 9203. J Clin Oncol (2006) 24(28):4620–5. doi: 10.1200/JCO.2006.06.7629 17008704

[4] Bosset JF Calais G Mineur L Maingon P Radosevic-Jelic L Daban A. Enhanced tumorocidal effect of chemotherapy with preoperative radiotherapy for rectal cancer: preliminary results–EORTC 22921. J Clin Oncol (2005) 23(24):5620–7. doi: 10.1200/JCO.2005.02.113 16009958

[5] Hofheinz RD Wenz F Post S Matzdorff A Laechelt S Hartmann JT. Chemoradiotherapy with capecitabine versus fluorouracil for locally advanced rectal cancer: a randomised, multicentre, non-inferiority, phase 3 trial. Lancet Oncol (2012) 13(6):579–88. doi: 10.1016/S1470-2045(12)70116-X 22503032

[6] Yuan Z Weng S Ye C Hu H Zhang S Yuan Y. CSCO guidelines for colorectal cancer version 2022: updates and discussions. Chin J Cancer Res (2022) 34(2):67–70. doi: 10.21147/j.issn.1000-9604.2022.02.01 35685997 PMC9086575

[7] Cedermark B Dahlberg M Glimelius B Påhlman L Rutqvist LE Wilking N. Improved survival with preoperative radiotherapy in resectable rectal cancer. N Engl J Med (1997) 336(14):980–7. doi: 10.1056/nejm199704033361402 9091798

[8] Martin ST Heneghan HM Winter DC. Systematic review and meta-analysis of outcomes following pathological complete response to neoadjuvant chemoradiotherapy for rectal cancer. Br J Surg (2012) 99(7):918–28. doi: 10.1002/bjs.8702 22362002

[9] Benson AB Venook AP Al-Hawary MM Arain MA Chen YJ Ciombor KK. Colon cancer, version 2.2021, NCCN clinical practice guidelines in oncology. J Natl Compr Cancer Netw (2021) 19(3):329–59. doi: 10.6004/jnccn.2021.0012 33724754

[10] Swedish Rectal Cancer Trial. Initial report from a Swedish multicentre study examining the role of preoperative irradiation in the treatment of patients with resectable rectal carcinoma. Br J Surg (1993) 80(10):1333–6. doi: 10.1002/bjs.1800801040 8242317

[11] Folkesson J Birgisson H Pahlman L Cedermark B Glimelius B Gunnarsson U. Swedish rectal cancer trial: long lasting benefits from radiotherapy on survival and local recurrence rate. J Clin Oncol (2005) 23(24):5644–50. doi: 10.1200/JCO.2005.08.144 16110023

[12] Kapiteijn E Marijnen CA Nagtegaal ID Putter H Steup WH Wiggers T. Preoperative radiotherapy combined with total mesorectal excision for resectable rectal cancer. N Engl J Med (2001) 345(9):638–46. doi: 10.1056/NEJMoa010580 11547717

[13] Van Gijn W Marijnen CA Nagtegaal ID Kranenbarg EM Putter H Wiggers T. Preoperative radiotherapy combined with total mesorectal excision for resectable rectal cancer: 12-year follow-up of the multicentre, randomised controlled TME trial. Lancet Oncol (2011) 12(6):575–82. doi: 10.1016/S1470-2045(11)70097-3 21596621

[14] Marijnen CA Kapiteijn E Van De Velde CJ Martijn H Steup WH Wiggers T. Acute side effects and complications after short-term preoperative radiotherapy combined with total mesorectal excision in primary rectal cancer: report of a multicenter randomized trial. J Clin Oncol (2002) 20(3):817–25. doi: 10.1200/JCO.2002.20.3.817 11821466

[15] Sebag-Montefiore D Stephens RJ Steele R Monson J Grieve R Khanna S. Preoperative radiotherapy versus selective postoperative chemoradiotherapy in patients with rectal cancer (MRC CR07 and NCIC-CTG C016): a multicentre, randomised trial. Lancet (2009) 373(9666):811–20. doi: 10.1016/S0140-6736(09)60484-0 PMC2668947 19269519

[16] Sauer R Becker H Hohenberger W Rödel C Wittekind C Fietkau R. Preoperative versus postoperative chemoradiotherapy for rectal cancer. N Engl J Med (2004) 351(17):1731–40. doi: 10.1056/NEJMoa040694 15496622

[17] Rödel C Graeven U Fietkau R Hohenberger W Hothorn T Arnold D. Oxaliplatin added to fluorouracil-based preoperative chemoradiotherapy and postoperative chemotherapy of locally advanced rectal cancer (the German CAO/ARO/AIO-04 study): final results of the multicentre, open-label, randomised, phase 3 trial. Lancet Oncol (2015) 16(8):979–89. doi: 10.1016/S1470-2045(15)00159-X 26189067

[18] Marijnen CA Nagtegaal ID Klein Kranenbarg E Hermans J Van De Velde CJ Leer JW. No downstaging after short-term preoperative radiotherapy in rectal cancer patients. J Clin Oncol (2001) 19(7):1976–84. doi: 10.1200/JCO.2001.19.7.1976 11283130

[19] Bujko K Nowacki MP Nasierowska-Guttmejer A Michalski W Bebenek M Pudełko M. Sphincter preservation following preoperative radiotherapy for rectal cancer: report of a randomised trial comparing short-term radiotherapy vs. conventionally fractionated radiochemotherapy. Radiother Oncol (2004) 72(1):15–24. doi: 10.1016/j.radonc.2003.12.006 15236870

[20] Bujko K Nowacki MP Nasierowska-Guttmejer A Michalski W Bebenek M Kryj M. Long-term results of a randomized trial comparing preoperative short-course radiotherapy with preoperative conventionally fractionated chemoradiation for rectal cancer. Br J Surg (2006) 93(10):1215–23. doi: 10.1002/bjs.5506 16983741

[21] Ngan SY Burmeister B Fisher RJ Solomon M Goldstein D Joseph D. Randomized trial of short-course radiotherapy versus long-course chemoradiation comparing rates of local recurrence in patients with T3 rectal cancer: trans-tasman radiation oncology group trial 01.04. J Clin Oncol (2012) 30(31):3827–33. doi: 10.1200/JCO.2012.42.9597 23008301

[22] Wang Y Shen L Wan J Zhang H Wu R Wang J. Neoadjuvant chemoradiotherapy combined with immunotherapy for locally advanced rectal cancer: a new era for anal preservation. Front Immunol (2022) 13:1067036. doi: 10.3389/fimmu.2022.1067036 36569918 PMC9772444

[23] Jakobsen A Mortensen JP Bisgaard C Lindebjerg J Hansen JW Rafaelsen SR. Preoperative chemoradiation of locally advanced T3 rectal cancer combined with an endorectal boost. Int J Radiat Oncol Biol Phys (2006) 64(2):461–5. doi: 10.1016/j.ijrobp.2005.07.969 16226396

[24] Jakobsen A Ploen J Vuong T Appelt A Lindebjerg J Rafaelsen SR. Dose-effect relationship in chemoradiotherapy for locally advanced rectal cancer: a randomized trial comparing two radiation doses. Int J Radiat Oncol Biol Phys (2012) 84(4):949–54. doi: 10.1016/j.ijrobp.2012.02.006 22592048

[25] Appelt AL Pløen J Vogelius IR Bentzen SM Jakobsen A. Radiation dose-response model for locally advanced rectal cancer after preoperative chemoradiation therapy. Int J Radiat Oncol Biol Phys (2013) 85(1):74–80. doi: 10.1016/j.ijrobp.2012.05.017 22763027 PMC3539741

[26] Appelt AL Pløen J Harling H Jensen FS Jensen LH Jørgensen JC. High-dose chemoradiotherapy and watchful waiting for distal rectal cancer: a prospective observational study. Lancet Oncol (2015) 16(8):919–27. doi: 10.1016/S1470-2045(15)00120-5 26156652

[27] Couwenberg AM Burbach JPM Berbee M Lacle MM Arensman R Raicu MG. Efficacy of dose-escalated chemoradiation on complete tumor response in patients with locally advanced rectal cancer (RECTAL-BOOST): a phase 2 randomized controlled trial. Int J Radiat Oncol Biol Phys (2020) 108(4):1008–18. doi: 10.1016/j.ijrobp.2020.06.013 32565319

[28] Bertocchi E Barugola G Nicosia L Mazzola R Ricchetti F Dell'Abate P. A comparative analysis between radiation dose intensification and conventional fractionation in neoadjuvant locally advanced rectal cancer: a monocentric prospective observational study. Radiol Med (2020) 125(10):990–8. doi: 10.1007/s11547-020-01189-9 32277332

[29] Wang J Guan Y Gu W Yan S Zhou J Huang D. Long-course neoadjuvant chemoradiotherapy with versus without a concomitant boost in locally advanced rectal cancer: a randomized, multicenter, phase II trial (FDRT-002). Radiat Oncol (2019) 14(1):215. doi: 10.1016/S0167-8140(19)30206-3 31783766 PMC6883699

[30] Abraham AG Joseph K Ghosh S Yun J Warkentin BJ Thai JJ. More is not better when it comes to treating rectal cancer with multimodal chemoradiation beyond the standard radiation dose of 5040 cGy. Dis Colon Rectum. (2022) 65(5):692–701. doi: 10.1097/DCR.0000000000001986 34082437

[31] Hearn N Atwell D Cahill K Elks J Vignarajah D Lagopoulos J. Neoadjuvant radiotherapy dose escalation in locally advanced rectal cancer: a systematic review and meta-analysis of modern treatment approaches and outcomes. Clin Oncol (R Coll Radiol). (2021) 33(1):e1–e14. doi: 10.1016/j.clon.2020.06.008 32669228

[32] Guido A Cuicchi D Castellucci P Cellini F Di Fabio F Llimpe FLR. Adaptive Individualized high-dose preoperAtive (AIDA) chemoradiation in high-risk rectal cancer: a phase II trial. Eur J Nucl Med Mol Imaging. (2023) 50(2):572–80. doi: 10.1007/s00259-022-05944-0 PMC9816267 36127416

[33] Carbonara R Surgo A Ciliberti MP Gregucci F Bonaparte I Nicosia L. Impact of preoperative chemoradiation with higher dose intensity modulated radiotherapy on pathological complete response for locally advanced rectal cancer: a systematic review. Expert Rev Anticancer Ther (2022) 22(11):1249–59. doi: 10.1080/14737140.2022.2130895 36174658

[34] Myint AS. Do nonoperative modality (NOM) treatments of rectal cancer compromise the chance of cure? Final surgical salvage results from OPERA phase 3 randomised trial (NCT02505750). J Clin Oncol (2023) 41(4_suppl):6. doi: 10.1200/JCO.2023.41.4_suppl.6

[35] Fiore M Greco C Coppola A Caricato M Caputo D Trecca P. Long-term results of a prospective phase 2 study on volume de-escalation in neoadjuvant chemoradiotherapy of rectal cancer. Pract Radiat Oncol (2021) 11(2):e186–e94. doi: 10.1016/j.prro.2020.09.009 33002647

[36] Song M Geng J Wang L Li Y Zhu X Li X. Excluding the ischiorectal fossa irradiation during neoadjuvant chemoradiotherapy with intensity-modulated radiotherapy followed by abdominoperineal resection decreases perineal complications in patients with lower rectal cancer. Radiat Oncol (2019) 14(1):138. doi: 10.1186/s13014-019-1338-5 31382984 PMC6683419

[37] Jin J Tang Y Hu C Jiang LM Jiang J Li N. Multicenter, randomized, phase III trial of short-term radiotherapy plus chemotherapy versus long-term chemoradiotherapy in locally advanced rectal cancer (STELLAR). J Clin Oncol (2022) 40(15):1681–92. doi: 10.1200/JCO.21.01667 PMC9113208 35263150

[38] Li S Zhang Y Yu Y Zhu X Geng J Teng H. Simultaneous integrated boost intensity-modulated radiation therapy can benefit the locally advanced rectal cancer patients with clinically positive lateral pelvic lymph node. Front Oncol (2020) 10:627572. doi: 10.3389/fonc.2020.627572 33692945 PMC7937798

[39] Chen M Liu S Xu M Yi HC Liu Y He F. Radiation boost for synchronous solitary inguinal lymph node metastasis during neoadjuvant chemoradiotherapy for locally advanced rectal cancer. Discovery Oncol (2021) 12(1):59. doi: 10.1007/s12672-021-00455-0 PMC8777535 35201468

[40] Zheng R Zhang Y Chen R Guan B Lin Y Wang B. Necessity of external iliac lymph nodes and inguinal nodes radiation in rectal cancer with anal canal involvement. BMC Cancer. (2022) 22(1):657. doi: 10.1186/s12885-022-09724-9 35701738 PMC9199347

[41] Song M Li S Zhang Y Geng J Wang H Zhu X. Is elective inguinal or external iliac irradiation during neoadjuvant (chemo)radiotherapy necessary for locally a dvanced lower rectal cancer with anal sphincter invasion? Pract Radiat Oncol (2022) 12(2):125–34. doi: 10.1016/j.prro.2021.10.003 34670136

[42] Basch E Dueck AC Mitchell SA Mamon H Weiser M Saltz L. Patient-reported outcomes during and after treatment for locally advanced rectal cancer in the PROSPECT trial (alliance N1048). J Clin Oncol (2023) 41(21):3724–34. doi: 10.1200/JCO.23.00903 PMC10351948 37270691

[43] Tsukada Y Kawashima Y Fujita F Koda K Ohue M Shinto E. Neoadjuvant chemotherapy (NAC) followed by total mesorectal excision (TME) and adjuvant chemotherapy versus TME followed by adjuvant chemotherapy in very low-lying clinical (c) T3 rectal cancer (NAIR): a multicenter, randomized, open-label, phase 2/3 trial. J Clin Oncol (2023) 41(16_suppl):3519. doi: 10.1200/JCO.2023.41.16_suppl.3519

[44] Azria D Doyen J Jarlier M Martel-Lafay I Hennequin C Etienne P. Late toxicities and clinical outcome at 5 years of the ACCORD 12/0405-PRODIGE 02 trial comparing two neoadjuvant chemoradiotherapy regimens for intermediate-risk rectal cancer. Ann Oncol (2017) 28(10):2436–42. doi: 10.1093/annonc/mdx351 28961836

[45] Allegra CJ Yothers G O'Connell MJ Beart RW Wozniak TF Pitot HC. Neoadjuvant 5-FU or capecitabine plus radiation with or without oxaliplatin in rectal cancer patients: a phase III randomized clinical trial. J Natl Cancer Inst (2015) 107(11):djv248. doi: 10.1093/jnci/djv248 26374429 PMC4849360

[46] Joybari AY Azadeh P Babaei S Kamal FH. Comparison of capecitabine (xeloda) vs. combination of capecitabine and oxaliplatin (XELOX) as neoadjuvant CRT for locally advanced rectal cancer. Pathol Oncol Res (2019) 25(4):1599–605. doi: 10.1007/s12253-019-00587-3 30712194

[47] Yang XH Li KG Wei JB Wu CH Liang SX Mo XW. Retrospective study of preoperative chemoradiotherapy with capecitabine versus capecitabine plus oxaliplatin for locally advanced rectal cancer. Sci Rep (2020) 10(1):12539. doi: 10.1038/s41598-020-69573-z 32719436 PMC7385078

[48] Valentini V Gambacorta MA Cellini F Aristei C Coco C Barbaro B. The INTERACT trial: long-term results of a randomised trial on preoperative capecitabine-based radiochemotherapy intensified by concomitant boost or oxaliplatin, for cT2 (distal)-cT3 rectal cancer. Radiother Oncol (2019) 134:110–8. doi: 10.1016/j.radonc.2018.11.023 31005204

[49] Kondo K Matsusaka S Ishihara S Horie H Uehara K Oguchi M. Long-term results of a multicenter phase II study of preoperative chemoradiotherapy with S-1 plus oxaliplatin for locally advanced rectal cancer (JACCRO CC-04: SHOGUN Trial). Radiother Oncol (2019) 134:199–203. doi: 10.1016/j.radonc.2019.02.006 31005216

[50] Deng Y Chi P Lan P Wang L Chen W Cui L. Neoadjuvant modified FOLFOX6 with or without radiation versus fluorouracil plus radiation for locally advanced rectal cancer: final results of the Chinese FOWARC trial. J Clin Oncol (2019) 37(34):3223–33. doi: 10.1200/JCO.18.02309 PMC6881102 31557064

[51] Chi P Lan P Cui L Wei H Zhao R Huang Z. Long-term outcome of neoadjuvant mFOLFOX6 with or without radiation versus fluorouracil plus radiation for locally advanced rectal cancer: a multicenter, randomized phase III trial. J Clin Oncol (2023) 41(16_suppl):3505. doi: 10.1200/JCO.2023.41.16_suppl.3505

[52] Hofheinz RD Von Gerstenberg-Helldorf B Wenz F Gnad U Kraus-Tiefenbacher U Müldner A. Phase I trial of capecitabine and weekly irinotecan in combination with radiotherapy for neoadjuvant therapy of rectal cancer. J Clin Oncol (2005) 23(7):1350–7. doi: 10.1200/JCO.2005.04.171 15684318

[53] Mehta VK Cho C Ford JM Jambalos C Poen J Koong A. Phase II trial of preoperative 3D conformal radiotherapy, protracted venous infusion 5-fluorouracil, and weekly CPT-11, followed by surgery for ultrasound-staged T3 rectal cancer. Int J Radiat Oncol Biol Phys (2003) 55(1):132–7. doi: 10.1016/S0360-3016(02)03863-4 12504045

[54] Klautke G Feyerherd P Ludwig K Prall F Foitzik T Fietkau R. Intensified concurrent chemoradiotherapy with 5-fluorouracil and irinotecan as neoadjuvant treatment in patients with locally advanced rectal cancer. Br J Cancer. (2005) 92(7):1215–20. doi: 10.1038/sj.bjc.6602492 PMC2361958 15785742

[55] Gollins S Myint AS Haylock B Wise M Saunders M Neupane R. Preoperative chemoradiotherapy using concurrent capecitabine and irinotecan in magnetic resonance imaging-defined locally advanced rectal cancer: impact on long-term clinical outcomes. J Clin Oncol (2011) 29(8):1042–9. doi: 10.1200/JCO.2010.29.7697 21263095

[56] Navarro M Dotor E Rivera F Sánchez-Rovira P Vega-Villegas ME Cervantes A. A phase II study of preoperative radiotherapy and concomitant weekly irinotecan in combination with protracted venous infusion 5-fluorouracil, for resectable locally advanced rectal cancer. Int J Radiat Oncol Biol Phys (2006) 66(1):201–5. doi: 10.1016/j.ijrobp.2006.04.007 16814947

[57] Mohiuddin M Winter K Mitchell E Hanna N Yuen A Nichols C. Randomized phase II study of neoadjuvant combined-modality chemoradiation for distal rectal cancer: radiation therapy oncology group trial 0012. J Clin Oncol (2006) 24(4):650–5. doi: 10.1200/JCO.2005.03.6095 16446336

[58] Glynne-Jones R Falk S Maughan TS Meadows HM Sebag-Montefiore D. A phase I/II study of irinotecan when added to 5-fluorouracil and leucovorin and pelvic radiation in locally advanced rectal cancer: a colorectal clinical oncology group study. Br J Cancer. (2007) 96(4):551–8. doi: 10.1038/sj.bjc.6603570 PMC2360056 17262086

[59] Zhu J Liu A Sun X Liu L Zhu Y Zhang T. Multicenter, randomized, phase III trial of neoadjuvant chemoradiation with capecitabine and irinotecan guided by UGT1A1 status in patients with locally advanced rectal cancer. J Clin Oncol (2020) 38(36):4231–9. doi: 10.1200/JCO.20.01932 PMC7768334 33119477

[60] Chau I Brown G Cunningham D Tait D Wotherspoon A Norman AR. Neoadjuvant capecitabine and oxaliplatin followed by synchronous chemoradiation and total mesorectal excision in magnetic resonance imaging-defined poor-risk rectal cancer. J Clin Oncol (2006) 24(4):668–74. doi: 10.1200/JCO.2005.04.4875 16446339

[61] Chua YJ Barbachano Y Cunningham D Oates JR Brown G Wotherspoon A. Neoadjuvant capecitabine and oxaliplatin before chemoradiotherapy and total mesorectal excision in MRI-defined poor-risk rectal cancer: a phase 2 trial. Lancet Oncol (2010) 11(3):241–8. doi: 10.1016/S1470-2045(09)70381-X 20106720

[62] Schou JV Larsen FO Rasch L Linnemann D Langhoff J Høgdall E. Induction chemotherapy with capecitabine and oxaliplatin followed by chemoradiotherapy before total mesorectal excision in patients with locally advanced rectal cancer. Ann Oncol (2012) 23(10):2627–33. doi: 10.1093/annonc/mds056 22473488

[63] Fernández-Martos C Pericay C Aparicio J Salud A Safont M Massuti B. randomized study of concomitant chemoradiotherapy followed by surgery and adjuvant capecitabine plus oxaliplatin (CAPOX) compared with induction CAPOX followed by concomitant chemoradiotherapy and surgery in magnetic resonance imaging-defined, locally advanced rectal cancer: grupo cancer de recto 3 study. J Clin Oncol (2010) 28(5):859–65. doi: 10.1200/JCO.2009.25.8541 20065174

[64] Maréchal R Vos B Polus M Delaunoit T Peeters M Demetter P. Short course chemotherapy followed by concomitant chemoradiotherapy and surgery in locally advanced rectal cancer: a randomized multicentric phase II study. Ann Oncol (2012) 23(6):1525–30. doi: 10.1093/annonc/mdr473 22039087

[65] Conroy T Bosset JF Etienne PL Rio E François É Mesgouez-Nebout N. Neoadjuvant chemotherapy with FOLFIRINOX and preoperative chemoradiotherapy for patients with locally advanced rectal cancer (UNICANCER-PRODIGE 23): a multicentre, randomised, open-label, phase 3 trial. Lancet Oncol (2021) 22(5):702–15. doi: 10.1016/S1470-2045(21)00079-6 33862000

[66] Etienne PL El R Evesque L Mesgouez-Nebout N Vendrely V Artignan X. Total neoadjuvant therapy with mFOLFIRINOX versus preoperative chemoradiation in patients with locally advanced rectal cancer: 7-year results of PRODIGE 23 phase III trial, a UNICANCER GI trial. J Clin Oncol (2023) 41(17_suppl):LBA3504. doi: 10.1200/JCO.2023.41.17_suppl.LBA3504 38986769

[67] Garcia-Aguilar J Chow OS Smith DD Marcet JE Cataldo PA Varma MG. Effect of adding mFOLFOX6 after neoadjuvant chemoradiation in locally advanced rectal cancer: a multicentre, phase 2 trial. Lancet Oncol (2015) 16(8):957–66. doi: 10.1016/S1470-2045(15)00004-2 PMC4670237 26187751

[68] Marco MR Zhou L Patil S Marcet JE Varma MG Oommen S. Consolidation mFOLFOX6 chemotherapy after chemoradiotherapy improves survival in patients with locally advanced rectal cancer: final results of a multicenter phase II Trial. Dis Colon Rectum. (2018) 61(10):1146–55. doi: 10.1097/DCR.0000000000001207 PMC6130918 30192323

[69] Zampino MG Magni E Leonardi MC Petazzi E Santoro L Luca F. Capecitabine initially concomitant to radiotherapy then perioperatively administered in locally advanced rectal cancer. Int J Radiat Oncol Biol Phys (2009) 75(2):421–7. doi: 10.1016/j.ijrobp.2008.11.002 19211200

[70] Zhu J Gu W Lian P Sheng W Cai G Shi D. A phase II trial of neoadjuvant IMRT-based chemoradiotherapy followed by one cycle of capecitabine for stage II/III rectal adenocarcinoma. Radiat Oncol (2013) 8:130. doi: 10.1186/1748-717X-8-130 23718210 PMC3680166

[71] Gao YH Zhang X An X Cai MY Zeng ZF Chen G. Oxaliplatin and capecitabine concomitant with neoadjuvant radiotherapy and extended to the resting period in high risk locally advanced rectal cancer. Strahlenther Onkol. (2014) 190(2):158–64. doi: 10.1007/s00066-013-0500-5 24408055

[72] Gao YH Lin JZ An X Luo JL Cai MY Cai PQ. Neoadjuvant sandwich treatment with oxaliplatin and capecitabine administered prior to, concurrently with, and following radiation therapy in locally advanced rectal cancer: a prospective phase 2 trial. Int J Radiat Oncol Biol Phys (2014) 90(5):1153–60. doi: 10.1016/j.ijrobp.2014.07.021 25442042

[73] Fokas E Allgäuer M Polat B Klautke G Grabenbauer GG Fietkau R. Randomized phase II trial of chemoradiotherapy plus induction or consolidation chemotherapy as total neoadjuvant therapy for locally advanced rectal cancer: CAO/ARO/AIO-12. J Clin Oncol (2019) 37(34):3212–22. doi: 10.1200/JCO.19.00308 31150315

[74] Kim SY Joo J Kim TW Hong YS Kim JE Hwang IG. A randomized phase 2 trial of consolidation chemotherapy after preoperative chemoradiation therapy versus chemoradiation therapy alone for locally advanced rectal cancer: KCSG CO 14-03. Int J Radiat Oncol Biol Phys (2018) 101(4):889–99. doi: 10.1016/j.ijrobp.2018.04.013 29976501

[75] Myerson RJ Tan B Hunt S Olsen J Birnbaum E Fleshman J. Five fractions of radiation therapy followed by 4 cycles of FOLFOX chemotherapy as preoperative treatment for rectal cancer. Int J Radiat Oncol Biol Phys (2014) 88(4):829–36. doi: 10.1016/j.ijrobp.2013.12.028 PMC4028157 24606849

[76] Markovina S Youssef F Roy A Aggarwal S Khwaja S DeWees T. Improved metastasis- and disease-free survival with preoperative sequential short-course radiation therapy and FOLFOX chemotherapy for rectal cancer compared with neoadjuvant long-course chemoradiotherapy: results of a matched pair analysis. Int J Radiat Oncol Biol Phys (2017) 99(2):417–26. doi: 10.1016/j.ijrobp.2017.05.048 28871992

[77] Bujko K Wyrwicz L Rutkowski A Malinowska M Pietrzak L Kryński J. Long-course oxaliplatin-based preoperative chemoradiation versus 5 × 5 Gy and consolidation chemotherapy for cT4 or fixed cT3 rectal cancer: results of a randomized phase III study. Ann Oncol (2016) 27(5):834–42. doi: 10.1093/annonc/mdw062 26884592

[78] Ciseł B Pietrzak L Michalski W Wyrwicz L Rutkowski A Kosakowska E. Long-course preoperative chemoradiation versus 5 × 5 Gy and consolidation chemotherapy for clinical T4 and fixed clinical T3 rectal cancer: long-term results of the randomized Polish II study. Ann Oncol (2019) 30(8):1298–303. doi: 10.1093/annonc/mdz186 31192355

[79] Chakrabarti D Rajan S Akhtar N Qayoom S Gupta S Verma M. Short-course radiotherapy with consolidation chemotherapy versus conventionally fractionated long-course chemoradiotherapy for locally advanced rectal cancer: randomized clinical trial. Br J Surg (2021) 108(5):511–20. doi: 10.1093/bjs/znab020 33724296

[80] Bahadoer RR Dijkstra EA Van Etten B Marijnen CAM Putter H Kranenbarg EM. Short-course radiotherapy followed by chemotherapy before total mesorectal excision (TME) versus preoperative chemoradiotherapy, TME, and optional adjuvant chemotherapy in locally advanced rectal cancer (RAPIDO): a randomised, open-label, phase 3 trial. Lancet Oncol (2021) 22(1):29–42. doi: 10.1016/S1470-2045(20)30555-6 33301740

[81] Dijkstra EA Nilsson PJ Hospers GAP Bahadoer RR Kranenbarg EMK Roodvoets AGH. Locoregional failure during and after short-course radiotherapy followed by chemotherapy and surgery compared to long-course chemoradiotherapy and surgery - a five-year follow-up of the RAPIDO trial. Ann Surg (2023) 278(4):e766–e772. doi: 10.1097/SLA.0000000000005799 36661037 PMC10481913

[82] Cercek A Roxburgh CSD Strombom P Smith JJ Temple LKF Nash GM. Adoption of total neoadjuvant therapy for locally advanced rectal cancer. JAMA Oncol (2018) 4(6):e180071. doi: 10.1001/jamaoncol.2018.0071 29566109 PMC5885165

[83] Chau I Allen M Cunningham D Tait D Brown G Hill M. Neoadjuvant systemic fluorouracil and mitomycin C prior to synchronous chemoradiation is an effective strategy in locally advanced rectal cancer. Br J Cancer. (2003) 88(7):1017–24. doi: 10.1038/sj.bjc.6600822 PMC2376366 12671697

[84] Cercek A Goodman KA Hajj C Weisberger E Segal NH Reidy-Lagunes DL. Neoadjuvant chemotherapy first, followed by chemoradiation and then surgery, in the management of locally advanced rectal cancer. J Natl Compr Cancer Netw (2014) 12(4):513–9. doi: 10.6004/jnccn.2014.0056 PMC5612781 24717570

[85] Chotard G Capdepont M Denost Q Smith D Vendrely V Rullier E. Effects of neoadjuvant chemotherapy plus chemoradiotherapy on lymph nodes in rectal adenocarcinoma. Virchows Arch (2021) 479(4):657–66. doi: 10.1007/s00428-021-03108-3 33983519

[86] Kim JK Marco MR Roxburgh CSD Chen CT Cercek A Strombom P. Survival after induction chemotherapy and chemoradiation versus chemoradiation and adjuvant chemotherapy for locally advanced rectal cancer. Oncologist (2022) 27(5):380–8. doi: 10.1093/oncolo/oyac025 PMC9074984 35278070

[87] Habr-Gama A Perez RO Sabbaga J Nadalin W Julião GPS Gama-Rodrigues J. Increasing the rates of complete response to neoadjuvant chemoradiotherapy for distal rectal cancer: results of a prospective study using additional chemotherapy during the resting period. Dis Colon Rectum. (2009) 52(12):1927–34. doi: 10.1007/DCR.0b013e3181ba14ed 19934911

[88] Cui J Dou X Sun Y Yue J. Consolidation chemotherapy may improve pathological complete response for locally advanced rectal cancer after neoadjuvant chemoradiotherapy: a retrospective study. PeerJ (2020) 8:e9513. doi: 10.7717/peerj.9513 32704453 PMC7350921

[89] Hatfield P Hingorani M Radhakrishna G Cooper R Melcher A Crellin A. Short-course radiotherapy, with elective delay prior to surgery, in patients with unresectable rectal cancer who have poor performance status or significant co-morbidity. Radiother Oncol (2009) 92(2):210–4. doi: 10.1016/j.radonc.2009.04.007 19409638

[90] Pettersson D Holm T Iversen H Blomqvist L Glimelius B Martling A. Preoperative short-course radiotherapy with delayed surgery in primary rectal cancer. Br J Surg (2012) 99(4):577–83. doi: 10.1002/bjs.7796 22241246

[91] Radu C Berglund A Påhlman L Glimelius B. Short-course preoperative radiotherapy with delayed surgery in rectal cancer - a retrospective study. Radiother Oncol (2008) 87(3):343–9. doi: 10.1016/j.radonc.2007.11.025 18093674

[92] Widder J Herbst F Scheithauer W. Preoperative sequential short-term radiotherapy plus chemotherapy can induce complete remission in T3N2 rectal cancer. Acta Oncol (2005) 44(8):921–3. doi: 10.1080/02841860500341199 16332603

[93] Romesser PB Park BK Nemirovsky D Alvarez J Omer DM Sarkar R. Organ preservation and total neoadjuvant therapy for rectal cancer: investigating longcourse chemoradiation versus short-course radiation therapy. J Clin Oncol (2023) 41(4):10. doi: 10.1200/JCO.2023.41.4_suppl.10

[94] Overman MJ Lonardi S Wong KYM Lenz HJ Gelsomino F Aglietta M. Durable clinical benefit with nivolumab plus ipilimumab in DNA mismatch repair-deficient/microsatellite instability-high metastatic colorectal cancer. J Clin Oncol (2018) 36(8):773–9. doi: 10.1200/JCO.2017.76.9901 29355075

[95] André T Shiu KK Kim TW Jensen BV Jensen LH Punt C. Pembrolizumab in microsatellite-instability-high advanced colorectal cancer. N Engl J Med (2020) 383(23):2207–18. doi: 10.1056/NEJMoa2017699 33264544

[96] Lugade AA Moran JP Gerber SA Rose RC Frelinger JG Lord EM. Local radiation therapy of B16 melanoma tumors increases the generation of tumor antigen-specific effector cells that traffic to the tumor. J Immunol (2005) 174(12):7516–23. doi: 10.4049/jimmunol.174.12.7516 15944250

[97] Victor CTS Rech AJ Maity A Rengan R Pauken KE Stelekati E. Radiation and dual checkpoint blockade activate non-redundant immune mechanisms in cancer. Nature (2015) 520(7547):373–7. doi: 10.1038/nature14292 PMC4401634 25754329

[98] Dovedi SJ Illidge TM. The antitumor immune response generated by fractionated radiation therapy may be limited by tumor cell adaptive resistance and can be circumvented by PD-L1 blockade. Oncoimmunology (2015) 4(7):e1016709. doi: 10.1080/2162402X.2015.1016709 26140246 PMC4485833

[99] Dovedi SJ Adlard AL Lipowska-Bhalla G McKenna C Jones S Cheadle EJ. Acquired resistance to fractionated radiotherapy can be overcome by concurrent PD-L1 blockade. Cancer Res (2014) 74(19):5458–68. doi: 10.1158/0008-5472.CAN-14-1258 25274032

[100] Bando H Tsukada Y Inamori K Togashi Y Koyama S Kotani D. Preoperative chemoradiotherapy plus nivolumab before surgery in patients with microsatellite stable and microsatellite instability-high locally advanced rectal cancer. Clin Cancer Res (2022) 28(6):1136–46. doi: 10.1158/1078-0432.CCR-21-3213 PMC9365382 35063964

[101] Tsukada Y Bando H Inamori K Wakabayashi M Togashi Y Koyama S. Survival outcomes and functional results of VOLTAGE-A: preoperative chemoradiotherapy (CRT) and consolidation nivolumab (nivo) in patients (pts) with both microsatellite stable (MSS) and microsatellite instability–high (MSI-H) locally advanced rectal cancer (LARC). J Clin Oncol (2023) 41(4_suppl):108. doi: 10.1200/JCO.2023.41.4_suppl.108

[102] George TJ Yothers G Jacobs SA Finley GG Wade JL Lima CMSPR. Phase II study of durvalumab following neoadjuvant chemoRT in operable rectal cancer: NSABP FR-2. J Clin Oncol (2022) 40(4 SUPPL):99. doi: 10.1200/JCO.2022.40.4_suppl.099

[103] Tamberi S Grassi E Zingaretti C Papiani G Pini S Corbelli J. A phase II study of capecitabine plus concomitant radiation therapy followed by durvalumab (MEDI4736) as preoperative treatment in rectal cancer: PANDORA study final results. J Clin Oncol (2022) 40(17):LBA3513. doi: 10.1200/JCO.2022.40.17_suppl.LBA3513

[104] Bensi M Salvatore L Corallo S Bergamo F Pellegrini I Prete AA. Phase ii study of preoperative (Preop) chemoradiotherapy (Ctrt) plus avelumab (Ave) in patients (Pts) with locally advanced rectal cancer (Larc): the avana study. Tumori (2021) 107(2 SUPPL):2. doi: 10.1200/JCO.2021.39.15_suppl.3511

[105] Carrasco J Schröder D Sinapi I De Cuyper A Beniuga G Delmarcelle S. R-IMMUNE interim analysis: a phase Ib/II study to evaluate safety and efficacy of atezolizumab combined with radio-chemotherapy in a preoperative setting for patients with localized rectal cancer. Ann Oncol (2021) 32:S537. doi: 10.1016/j.annonc.2021.08.919

[106] Rahma OE Yothers G Hong TS Russell MM You YN Parker W. Use of total neoadjuvant therapy for locally advanced rectal cancer: initial results from the pembrolizumab arm of a phase 2 randomized clinical trial. JAMA Oncol (2021) 7(8):1225–30. doi: 10.1001/jamaoncol.2021.1683 PMC8251652 34196693

[107] George TJ Yothers G Rahma OE Hong TS Russell MM You YN. Long-term results from NRG-GI002: a phase II clinical trial platform using total neoadjuvant therapy (TNT) in locally advanced rectal cancer (LARC). J Clin Oncol (2023) 41(4_suppl):7. doi: 10.1200/JCO.2023.41.4_suppl.7 36343307

[108] Zhou L Yu G Shen Y Ding H Zheng K Wen R. The clinical efficacy and safety of neoadjuvant chemoradiation therapy with immunotherapy for the organ preservation of ultra low rectal cancer: a single arm and open label exploratory study. J Clin Oncol (2022) 40(16):e15603. doi: 10.1200/JCO.2022.40.16_suppl.e15603

[109] Wu A Li Y Ji D Zhang L Zhang X Cai Y. PKUCH 04 trial: total neoadjuvant chemoradiation combined with neoadjuvant PD-1 blockade for pMMR/MSS locally advanced middle to low rectal cancer. J Clin Oncol (2022) 40(16):3609. doi: 10.1200/JCO.2022.40.16_suppl.3609

[110] Yao H Yang Z Gao J Zhang X Wu G Wei D. Safety and efficacy evaluation of long course neoadjuvant chemoradiotherapy plus tislelizumab followed by total mesorectal excision for locally advanced rectal cancer: short-term results of a multicenter, phase II study. J Clin Oncol (2022) 40(16):e15599. doi: 10.1200/JCO.2022.40.16_suppl.e15599 PMC9044580 35477432

[111] Lin Z Cai M Zhang P Li G Liu T Li X. single-arm trial of preoperative short-course radiotherapy followed by chemotherapy and camrelizumab in locally advanced rectal cancer. J Immunother Cancer. (2021) 9(11):e003554. doi: 10.1136/jitc-2021-003554 34725214 PMC8562535

[112] Shamseddine A Zeidan YH El Husseini Z Kreidieh M Al Darazi M Turfa R. Efficacy and safety-in analysis of short-course radiation followed by mFOLFOX-6 plus avelumab for locally advanced rectal adenocarcinoma. Radiat Oncol (2020) 15(1):233. doi: 10.1186/s13014-020-01673-6 33028346 PMC7542723

[113] Chalabi M Fanchi LF Dijkstra KK Van den Berg JG Aalbers AG Sikorska K. Neoadjuvant immunotherapy leads to pathological responses in MMR-proficient and MMR-deficient early-stage colon cancers. Nat Med (2020) 26(4):566–76. doi: 10.1038/s41591-020-0805-8 32251400

[114] Hu H Kang L Zhang J Wu Z Wang H Huang M. Neoadjuvant PD-1 blockade with toripalimab, with or without celecoxib, in mismatch repair-deficient or microsatellite instability-high, locally advanced, colorectal cancer (PICC): a single-centre, parallel-group, non-comparative, randomised, phase 2 trial. Lancet Gastroenterol Hepatol (2022) 7(1):38–48. doi: 10.1016/S2468-1253(21)00348-4 34688374

[115] Cercek A Lumish MA Sinopoli JC Weiss JA Shia J Stadler ZK. Single agent PD-1 blockade as curative-intent treatment in mismatch repair deficient locally advanced rectal cancer. J Clin Oncol (2022) 40(17_suppl):LBA5–LBA. doi: 10.1200/JCO.2022.40.17_suppl.LBA5 PMC9492301 35660797

[116] Yang R Wu T Yu J Cai X Li G Li X. Locally advanced rectal cancer with dMMR/MSI-H may be excused from surgery after neoadjuvant anti-PD-1 monotherapy: a multiple-center, cohort study. Front Immunol (2023) 14:1182299. doi: 10.3389/fimmu.2023.1182299 37441082 PMC10333582

[117] Chen G Jin Y Guan WL Zhang RX Xiao WW Cai PQ. Neoadjuvant PD-1 blockade with sintilimab in mismatch-repair deficient, locally advanced rectal cancer: an open-label, single-centre phase 2 study. Lancet Gastroenterol Hepatol (2023) 8(5):422–31. doi: 10.1016/S2468-1253(22)00439-3 36870360

[118] Wang Y Shen L Wan J Zhang H Wu R Wang J. Short-course radiotherapy combined with CAPOX and Toripalimab for the total neoadjuvant therapy of locally advanced rectal cancer: a randomized, prospective, multicentre, double-arm, phase II trial (TORCH). BMC Cancer. (2022) 22(1):274. doi: 10.1186/s12885-022-09348-z 35291966 PMC8922781

[119] Dai S Wang F Shen Y Shi L Xu L Lai C. Efficacy and safety of neoadjuvant preoperative short-course radiation followed by envafolimab plus CAPEOX in microsatellite stable (MSS)/mismatch repair proficient (pMMR) locally advanced rectal cancer. J Clin Oncol (2023) 41(4_suppl):134. doi: 10.1200/JCO.2023.41.4_suppl.134

[120] Li S Kim JS Kim JM Cho MJ Yoon WH Song KS. Epidermal growth factor receptor as a prognostic factor in locally advanced rectal-cancer patients treated with preoperative chemoradiation. Int J Radiat Oncol Biol Phys (2006) 65(3):705–12. doi: 10.1016/j.ijrobp.2006.01.013 16690221

[121] Bertolini F Bengala C Losi L Pagano M Iachetta F Dealis C. Prognostic and predictive value of baseline and posttreatment molecular marker expression in locally advanced rectal cancer treated with neoadjuvant chemoradiotherapy. Int J Radiat Oncol Biol Phys (2007) 68(5):1455–61. doi: 10.1016/j.ijrobp.2007.02.018 17445998

[122] Willett CG Duda DG Di Tomaso E Boucher Y Ancukiewicz M Sahani DV. Efficacy, safety, and biomarkers of neoadjuvant bevacizumab, radiation therapy, and fluorouracil in rectal cancer: a multidisciplinary phase II study. J Clin Oncol (2009) 27(18):3020–6. doi: 10.1200/JCO.2008.21.1771 PMC2702234 19470921

[123] Crane CH Eng C Feig BW Das P Skibber JM Chang GJ. Phase II trial of neoadjuvant bevacizumab, capecitabine, and radiotherapy for locally advanced rectal cancer. Int J Radiat Oncol Biol Phys (2010) 76(3):824–30. doi: 10.1016/j.ijrobp.2009.02.037 19464823

[124] Yu X Wang QX Xiao WW Chang H Zeng ZF Lu ZH. Neoadjuvant oxaliplatin and capecitabine combined with bevacizumab plus radiotherapy for locally advanced rectal cancer: results of a single-institute phase II study. Cancer Commun (Lond). (2018) 38(1):24. doi: 10.1186/s40880-018-0294-z 29784042 PMC5993137

[125] Velenik V Ocvirk J Music M Bracko M Anderluh F Oblak I. Neoadjuvant capecitabine, radiotherapy, and bevacizumab (CRAB) in locally advanced rectal cancer: results of an open-label phase II study. Radiat Oncol (2011) 6:105. doi: 10.1186/1748-717X-6-105 21880132 PMC3179720

[126] Gasparini G Torino F Ueno T Cascinu S Troiani T Ballestrero A. A phase II study of neoadjuvant bevacizumab plus capecitabine and concomitant radiotherapy in patients with locally advanced rectal cancer. Angiogenesis (2012) 15(1):141–50. doi: 10.1007/s10456-011-9250-0 22212406

[127] Landry JC Feng Y Cohen SJ Staley CA Whittington R Sigurdson ER. Phase 2 study of preoperative radiation with concurrent capecitabine, oxaliplatin, and bevacizumab followed by surgery and postoperative 5-fluorouracil, leucovorin, oxaliplatin (FOLFOX), and bevacizumab in patients with locally advanced rectal cancer: ECOG 3204. Cancer (2013) 119(8):1521–7. doi: 10.1002/cncr.27890 PMC3620731 23288663

[128] Dellas K Höhler T Reese T Würschmidt F Engel E Rödel C. Phase II trial of preoperative radiochemotherapy with concurrent bevacizumab, capecitabine and oxaliplatin in patients with locally advanced rectal cancer. Radiat Oncol (2013) 8:90. doi: 10.1186/1748-717X-8-90 23587311 PMC3679876

[129] Borg C André T Mantion G Boudghène F Mornex F Maingon P. Pathological response and safety of two neoadjuvant strategies with bevacizumab in MRI-defined locally advanced T3 resectable rectal cancer: a randomized, noncomparative phase II study. Ann Oncol (2014) 25(11):2205–10. doi: 10.1093/annonc/mdu377 25122693

[130] Masi G Vivaldi C Fornaro L Lonardi S Buccianti P Sainato A. Total neoadjuvant approach with FOLFOXIRI plus bevacizumab followed by chemoradiotherapy plus bevacizumab in locally advanced rectal cancer: the TRUST trial. Eur J Cancer. (2019) 110:32–41. doi: 10.1016/j.ejca.2019.01.006 30739838

[131] Marti FEM Jayson GC Manoharan P O'Connor J Renehan AG Backen AC. Novel phase I trial design to evaluate the addition of cediranib or selumetinib to preoperative chemoradiotherapy for locally advanced rectal cancer: the DREAMtherapy trial. Eur J Cancer. (2019) 117:48–59. doi: 10.1016/j.ejca.2019.04.029 31229949

[132] Bertolini F Chiara S Bengala C Antognoni P Dealis C Zironi S. Neoadjuvant treatment with single-agent cetuximab followed by 5-FU, cetuximab, and pelvic radiotherapy: a phase II study in locally advanced rectal cancer. Int J Radiat Oncol Biol Phys (2009) 73(2):466–72. doi: 10.1016/j.ijrobp.2008.04.065 19004567

[133] Horisberger K Treschl A Mai S Barreto-Miranda M Kienle P Ströbel P. Cetuximab in combination with capecitabine, irinotecan, and radiotherapy for patients with locally advanced rectal cancer: results of a Phase II MARGIT trial. Int J Radiat Oncol Biol Phys (2009) 74(5):1487–93. doi: 10.1016/j.ijrobp.2008.10.014 19131187

[134] Kim SY Hong YS Kim DY Kim TW Kim JH Im SA. Preoperative chemoradiation with cetuximab, irinotecan, and capecitabine in patients with locally advanced resectable rectal cancer: a multicenter phase II study. Int J Radiat Oncol Biol Phys (2011) 81(3):677–83. doi: 10.1016/j.ijrobp.2010.06.035 20888703

[135] Dewdney A Cunningham D Tabernero J Capdevila J Glimelius B Cervantes A. Multicenter randomized phase II clinical trial comparing neoadjuvant oxaliplatin, capecitabine, and preoperative radiotherapy with or without cetuximab followed by total mesorectal excision in patients with high-risk rectal cancer (EXPERT-C). J Clin Oncol (2012) 30(14):1620–7. doi: 10.1200/JCO.2011.39.6036 22473163

[136] Valentini V De Paoli A Gambacorta MA Mantini G Ratto C Vecchio FM. Infusional 5-fluorouracil and ZD1839 (Gefitinib-Iressa) in combination with preoperative radiotherapy in patients with locally advanced rectal cancer: a phase I and II Trial (1839IL/0092). Int J Radiat Oncol Biol Phys (2008) 72(3):644–9. doi: 10.1016/j.ijrobp.2008.01.046 18395356

[137] Pinto C Di Fabio F Maiello E Pini S Latiano T Aschele C. Phase II study of panitumumab, oxaliplatin, 5-fluorouracil, and concurrent radiotherapy as preoperative treatment in high-risk locally advanced rectal cancer patients (StarPan/STAR-02 Study). Ann Oncol (2011) 22(11):2424–30. doi: 10.1093/annonc/mdq782 21385884

[138] Pinto C Di Bisceglie M Di Fabio F Bochicchio A Latiano T Cordio S. Phase II study of preoperative treatment with external radiotherapy plus panitumumab in low-risk, locally advanced rectal cancer (RaP Study/STAR-03). Oncologist (2018) 23(8):912–8. doi: 10.1634/theoncologist.2017-0484 PMC6156174 29523646

[139] Helbling D Bodoky G Gautschi O Sun H Bosman F Gloor B. Neoadjuvant chemoradiotherapy with or without panitumumab in patients with wild-type KRAS, locally advanced rectal cancer (LARC): a randomized, multicenter, phase II trial SAKK 41/07. Ann Oncol (2013) 24(3):718–25. doi: 10.1093/annonc/mds519 23139259

[140] Presta LG Chen H O'Connor SJ Chisholm V Meng YG Krummen L. Humanization of an anti-vascular endothelial growth factor monoclonal antibody for the therapy of solid tumors and other disorders. Cancer Res (1997) 57(20):4593–9. 9377574

[141] Willett CG Duda DG Ancukiewicz M Shah M Czito BG Bentley R. A safety and survival analysis of neoadjuvant bevacizumab with standard chemoradiation in a phase I/II study compared with standard chemoradiation in locally advanced rectal cancer. Oncologist (2010) 15(8):845–51. doi: 10.1634/theoncologist.2010-0030 PMC3078712 20667969

[142] Koukourakis MI Giatromanolaki A Tsoutsou P Lyratzopoulos N Pitiakoudis M Kouklakis G. Bevacizumab, capecitabine, amifostine, and preoperative hypofractionated accelerated radiotherapy (HypoArc) for rectal cancer: a phase II study. Int J Radiat Oncol Biol Phys (2011) 80(2):492–8. doi: 10.1016/j.ijrobp.2010.02.037 20584585

[143] Kennecke H Berry S Wong R Zhou C Tankel K Easaw J. Pre-operative bevacizumab, capecitabine, oxaliplatin and radiation among patients with locally advanced or low rectal cancer: a phase II trial. Eur J Cancer. (2012) 48(1):37–45. doi: 10.1016/j.ejca.2011.05.016 21664123

[144] Spigel DR Bendell JC McCleod M Shipley DL Arrowsmith E Barnes EK. Phase II study of bevacizumab and chemoradiation in the preoperative or adjuvant treatment of patients with stage II/III rectal cancer. Clin Colorectal Cancer. (2012) 11(1):45–52. doi: 10.1016/j.clcc.2011.04.002 21840771

[145] Sadahiro S Suzuki T Tanaka A Okada K Saito G Kamijo A. Phase II study of preoperative concurrent chemoradiotherapy with S-1 plus bevacizumab for locally advanced resectable rectal adenocarcinoma. Oncology (2015) 88(1):49–56. doi: 10.1159/000367972 25277532

[146] Higashijima J Tokunaga T Yoshimoto T Eto S Kashihara H Takasu C. A multicenter phase II trial of preoperative chemoradiotherapy with S-1 plus oxaliplatin and bevacizumab for locally advanced rectal cancer. Int J Clin Oncol (2021) 26(5):875–82. doi: 10.1007/s10147-021-01868-1 33486623

[147] Dipetrillo T Pricolo V Lagares-Garcia J Vrees M Klipfel A Cataldo T. Neoadjuvant bevacizumab, oxaliplatin, 5-fluorouracil, and radiation for rectal cancer. Int J Radiat Oncol Biol Phys (2012) 82(1):124–9. doi: 10.1016/j.ijrobp.2010.08.005 20947267

[148] Resch G De Vries A Öfner D Eisterer W Rabl H Jagoditsch M. Preoperative treatment with capecitabine, bevacizumab and radiotherapy for primary locally advanced rectal cancer–a two stage phase II clinical trial. Radiother Oncol (2012) 102(1):10–3. doi: 10.1016/j.radonc.2011.06.008 21741716

[149] Akiyoshi T Shinozaki E Taguchi S Chino A Hiratsuka M Tominaga T. Non-operative management after chemoradiotherapy plus consolidation or sandwich (induction with bevacizumab and consolidation) chemotherapy in patients with locally advanced rectal cancer: a multicentre, randomised phase II trial (NOMINATE trial). BMJ Open (2022) 12(3):e055140. doi: 10.1136/bmjopen-2021-055140 PMC8935173 35304396

[150] Verschoor YL Lambregts DMJ Van Den Berg J Grotenhuis BA Aalbers A Van Triest B. Radiotherapy, atezolizumab, and bevacizumab in rectal cancers with the aim of organ preservation: the TARZAN study. J Clin Oncol (2023) 41(4_suppl):158. doi: 10.1200/JCO.2023.41.4_suppl.158

[151] Bonner JA Maihle NJ Folven BR Christianson TJ Spain K. The interaction of epidermal growth factor and radiation in human head and neck squamous cell carcinom a cell lines with vastly different radiosensitivities. Int J Radiat Oncol Biol Phys (1994) 29(2):243–7. doi: 10.1016/0360-3016(94)90269-0 8195014

[152] Ping Y Jian Z Yi Z Huoyu Z Feng L Yuqiong Y. Inhibition of the EGFR with nanoparticles encapsulating antisense oligonucleotides of the EGFR enhances radiosensitivity in SCCVII cells. Med Oncol (2010) 27(3):715–21. doi: 10.1007/s12032-009-9274-0 19653138

[153] Rödel C Arnold D Hipp M Liersch T Dellas K Iesalnieks I. Phase I-II trial of cetuximab, capecitabine, oxaliplatin, and radiotherapy as preoperative treatment in rectal cancer. Int J Radiat Oncol Biol Phys (2008) 70(4):1081–6. doi: 10.1016/j.ijrobp.2007.07.2356 17881150

[154] Kim SY Shim EK Yeo HY Baek JY Hong YS Kim DY. KRAS mutation status and clinical outcome of preoperative chemoradiation with cetuximab in locally advanced rectal cancer: a pooled analysis of 2 phase II trials. Int J Radiat Oncol Biol Phys (2013) 85(1):201–7. doi: 10.1016/j.ijrobp.2012.03.048 22672749

[155] Velenik V Ocvirk J Oblak I Anderluh F. A phase II study of cetuximab, capecitabine and radiotherapy in neoadjuvant treatment of patients with locally advanced resectable rectal cancer. Eur J Surg Oncol (2010) 36(3):244–50. doi: 10.1016/j.ejso.2009.12.002 20042310

[156] Marijnen CA Van De Velde CJ Putter H Van Den Brink M Maas CP Martijn H. Impact of short-term preoperative radiotherapy on health-related quality of life and sexual functioning in primary rectal cancer: report of a multicenter randomized trial. J Clin Oncol (2005) 23(9):1847–58. doi: 10.1200/JCO.2005.05.256 15774778

[157] Bujko K Nowacki MP Kepka L Oledzki J Bebenek M Kryj M. Postoperative complications in patients irradiated pre-operatively for rectal cancer: report of a randomised trial comparing short-term radiotherapy vs chemoradiation. Colorectal Dis (2005) 7(4):410–6. doi: 10.1111/j.1463-1318.2005.00796.x 15932569

[158] Fernandez-Martos C Garcia-Albeniz X Pericay C Maurel J Aparicio J Montagut C. Chemoradiation, surgery and adjuvant chemotherapy versus induction chemotherapy followed by chemoradiation and surgery: long-term results of the Spanish GCR-3 phase II randomized trial†. Ann Oncol (2015) 26(8):1722–8. doi: 10.1093/annonc/mdv223 25957330

[159] Fokas E Schlenska-Lange A Polat B Klautke G Grabenbauer GG Fietkau R. Chemoradiotherapy plus induction or consolidation chemotherapy as total neoadjuvant therapy for patients with locally advanced rectal cancer: long-term results of the CAO/ARO/AIO-12 randomized clinical trial. JAMA Oncol (2022) 8(1):e215445. doi: 10.1001/jamaoncol.2021.5445 34792531 PMC8603234

[160] Pettersson D Lörinc E Holm T Iversen H Cedermark B Glimelius B. Tumour regression in the randomized Stockholm III Trial of radiotherapy regimens for rectal cancer. Br J Surg (2015) 102(8):972–8. doi: 10.1002/bjs.9811 PMC4744683 26095256

[161] Borg C Mantion G Boudghène F Mornex F Ghiringhelli F Adenis A. Efficacy and safety of two neoadjuvant strategies with bevacizumab in MRI-defined locally advanced T3 resectable rectal cancer: final results of a randomized, noncomparative phase 2 INOVA study. Clin Colorectal Cancer. (2019) 18(3):200–8.e1. doi: 10.1016/j.clcc.2019.04.006 31311761

